# Rap1 regulates hematopoietic stem cell survival and affects oncogenesis and response to chemotherapy

**DOI:** 10.1038/s41467-019-13082-9

**Published:** 2019-12-13

**Authors:** Ekta Khattar, Kyaw Ze Ya Maung, Chen Li Chew, Arkasubhra Ghosh, Michelle Meng Huang Mok, Pei Lee, Jun Zhang, Wei Hong Jeff Chor, Gökhan Cildir, Chelsia Qiuxia Wang, Nur Khairiah Mohd-Ismail, Desmond Wai Loon Chin, Soo Chin Lee, Henry Yang, Yong-Jae Shin, Do-Hyun Nam, Liming Chen, Alan Prem Kumar, Lih Wen Deng, Masahito Ikawa, Jayantha Gunaratne, Motomi Osato, Vinay Tergaonkar

**Affiliations:** 1 Institute of Molecular and Cell Biology (IMCB), Agency for Science, Technology and Research (A-STAR), Singapore, Singapore; 2 Cancer Science Institute of Singapore, National University of Singapore, Singapore, Singapore; 3 Department of Biochemistry, Yong Loo Lin School of Medicine, National University of Singapore, Singapore, Singapore; 4 Jiangsu Key Laboratory for Molecular and Medical Biotechnology, College of Life Science, Nanjing Normal University, 210023 Nanjing, P.R. China; 5 Institute for Refractory Cancer Research, Samsung Medical Center, Seoul, Republic of Korea; 6 Research Institute for Microbial Diseases, Osaka University, Suita, Osaka 565-0871 Japan; 7 Graduate School of Medicine, Osaka University, Suita, Osaka 565-0871 Japan; 8 Department of Pathology, Yong Loo Lin School of Medicine, National University of Singapore, Singapore, Singapore

**Keywords:** Cancer, Cell biology, Molecular biology, Stem cells, Oncology

## Abstract

Increased levels and non-telomeric roles have been reported for shelterin proteins, including RAP1 in cancers. Herein using Rap1 null mice, we provide the genetic evidence that mammalian Rap1 plays a major role in hematopoietic stem cell survival, oncogenesis and response to chemotherapy. Strikingly, this function of RAP1 is independent of its association with the telomere or with its known partner TRF2. We show that RAP1 interacts with many members of the DNA damage response (DDR) pathway. RAP1 depleted cells show reduced interaction between XRCC4/DNA Ligase IV and DNA-PK, and are impaired in DNA Ligase IV recruitment to damaged chromatin for efficient repair. Consistent with its role in DNA damage repair, RAP1 loss decreases double-strand break repair via NHEJ in vivo, and consequently reduces B cell class switch recombination. Finally, we discover that RAP1 levels are predictive of the success of chemotherapy in breast and colon cancer.

## Abstract (web summary)

Rap1 is a telomeric protein that is highly expressed in cancers. Here, the authors show that Rap1 interacts with several DNA repair proteins independent of its telomere function to enhance DNA repair and that its deficiency leads to accelerated tumorigenesis, but enhanced sensitivity to genotoxic stress.

## Introduction

RAP1 (also known as TERF2IP) is a member of the shelterin complex that associates with and protects mammalian telomeres^1,2^. This complex is composed of five other core members, namely TRF1, TRF2, TIN2, POT1, and TPP1, and it is also important for regulating telomerase recruitment^3^. Human RAP1 was identified as a TRF2-interacting protein that is an ortholog of the yeast telomeric protein Rap1p^4^. Multiple roles have been described for yeast Rap1p both at the telomere and at other sites on the chromatin, including telomere length regulation, transcriptional activation, heterochromatin boundary-element formation, and subtelomeric silencing through recruitment of the Sir complex^3^. Human RAP1 differs from its yeast counterpart in that it is unable to directly bind telomeric DNA and it requires interaction with TRF2 for recruitment to the telomeres^4^. There is recent evidence suggesting that human RAP1 may interact directly with DNA in a sequence-independent manner^5^. Similar to yeast Rap1p, mammalian Rap1 has been suggested to play a role in telomere length maintenance^6,7^. However, recent studies have revealed that mammalian Rap1 is dispensable for telomere capping, indicating that Rap1 may instead protect telomeres indirectly through the regulation of DNA repair activities at telomere ends^8–12^. Sfeir et al.^9^ reported that the conditional loss of Rap1 alone did not affect telomere length, induce the formation of telomere dysfunction-induced foci (TIFs) or promote telomeric fusions. The roles of mammalian Rap1 are only beginning to be clearly understood.

Since increased levels of RAP1 and other telomeric proteins are seen in many human malignancies, understanding their telomeric and non-telomeric roles has gained momentum^3,13–18^. Non-canonical roles were first described for TRF2 and TIN2 at non-telomeric DNA double-strand breaks (DSB) and heterochromatin protein 1γ-marked extra-telomeric sites, respectively^19,20^. Recently, using genome-wide chromatin immunoprecipitation-sequencing (ChIP-seq), RAP1 was shown to regulate gene transcription^12^. Additionally, TPP1, RAP1, POT1, and TIN2 were shown to be present in non-chromatin-bound cellular fractions, suggesting a broader role for these proteins away from the telomeres^13,21^. In an unbiased screen for regulators of nuclear factor κB (NFκB), we reported that RAP1 promotes NFκB-dependent inflammatory gene expression^22^. Our results demonstrates a telomere-independent, cytoplasmic role for mammalian Rap1. Rap1 has also previously been shown to have extra-telomeric functions in transcriptional regulation of metabolic pathways^23,24^. However, the possible roles of RAP1 in regulating signaling pathways which influence cancer initation, progression, or treatment are largely unknown.

There have been reports of interactions between RAP1 and members of the DNA damage response (DDR) pathway^7,25^. However, the functional significance of these interactions remains to be elucidated. Considering the well-established role of DDR in genome stability and cancer prevention, investigation into the role of RAP1 in the context of DNA repair and tumorigenesis is an important unexplored area. In this report we uncover that mammalian Rap1 interacts with several DNA repair proteins independent of TRF2 and functions as an adapter for key proteins which mediate repair in response to DNA damage initated by genotoxic agents and oncogenes. Most notably, the importance for the role of RAP1 in DDR is provided by data from human clinical trials which shows that RAP1 levels correlate with the success of chemotherapy for breast and colon cancer using DNA damaging agents. Overall, these findings have implications for initiation, progression and treatment of cancer.

## Results

### Rap1-deficient mice do not display gross abnormalities

In order to genetically evaluate the role of Rap1 in mammals, we generated Rap1 conditional knockout mice. Exon 2 of *Rap1* flanked by loxP sites was inserted into the *Rap1* locus (Supplementary Fig. 1A). To generate whole-body *Rap1* knockout mice (KO, *Rap1*^−/−^), chimeric mice inheriting this recombinant *Rap1* allele were crossed to β-actin-Cre transgenic mice^26^. Mouse embryonic fibroblasts (MEFs) derived from E13.5 embryos confirmed the loss of Rap1 protein and mRNA expression in knockout cells (Supplementary Fig. 1B). *Rap1* KO mice were born at normal Mendelian frequencies, were fertile, and showed no overt pathologies at birth. As previously reported, Rap1 null mice were obese and this phenotype was particularly evident in females^23,24^.

Bone marrow (BM) failure is a characteristic feature of several diseases known to be associated with telomere dysfunction^27^. To test the role of Rap1 in BM homeostasis in mice, we evaluated the status of different hematopoietic cells with or without Rap1 at steady-state. Hematological parameters such as white blood cells, hemoglobin, and platelet counts between *Rap1* wild type (WT) and KO mice (Supplementary Fig. 1C) did not reveal any significant differences. The percentage of myeloid progenitors (c-Kit^+^ Sca-1^-^ Lin^-^, KL, where c-Kit^+^ denotes c-Kit-positive cells, Sca-1^−^ denotes Sca-1-negative cells) and Hematopoietic Stem and Progenitor Cells (HSPC, c-Kit^+^ Sca-1^+^ Lin^−^, KSL) in the BM were similar in both *Rap1* WT and KO mice (Supplementary Fig. 1D, E). Furthermore, populations of myeloid and erythroid cells in the BM and T cells and B cells in the spleen were comparable between *Rap1* KO mice and WT controls (Supplementary Fig. 1F). A detailed analysis of the T cells and B cells at various stages of development and maturity show no significant differences between *Rap1* WT and *Rap1* KO mice (Supplementary Fig. 1G, H). Thus, under homeostasis, the loss of Rap1 expression does not have any significant effects on the different hematopoietic cell populations in the BM and spleen.

### Loss of Rap1 sensitizes mice to DNA damaging agents

**Fig. 1:**
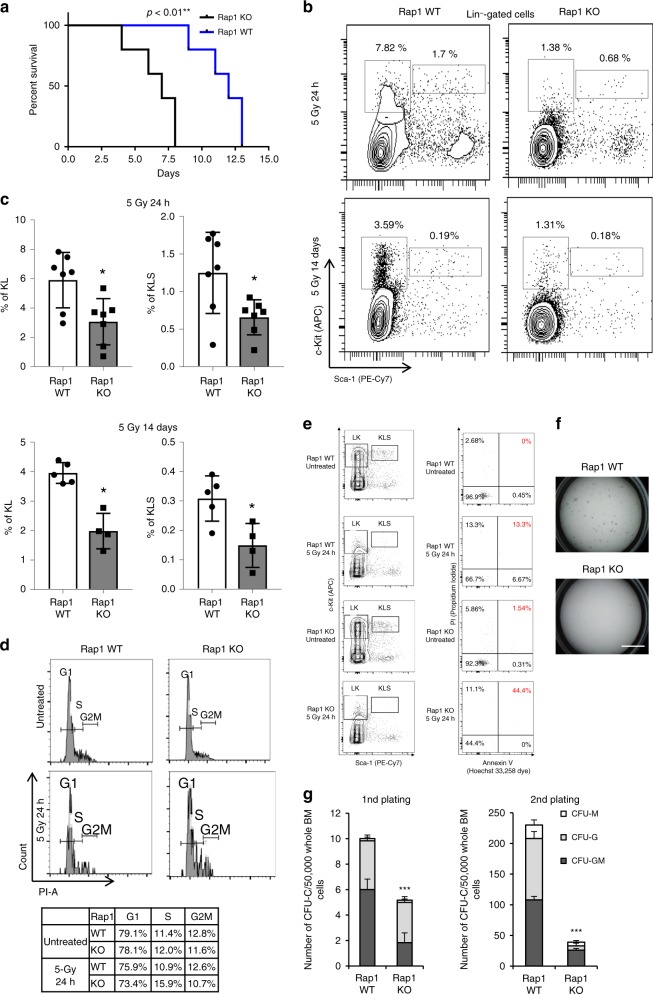
Loss of Rap1 sensitizes mice to γ-irradiation and 5-FU. **a** Survival curve for *Rap1* WT and KO mice treated with 11 Gy γ-irradiation (*n* = 5/genotype). **b** Flow cytometric analysis of HSPC compartment in bone marrow (BM) of 6- to 8-week-old mice 24 h and 14 days after a single dose of 5 Gy irradiation. Representative FACS plots of 200,000 cells gated on viable Lin^-^ cells are shown. Percentages in parent gate are shown. **c** Graphical representation of the results presented in **b** is shown. Mean ± SEM of the percentage of KSL (right) and KL (left) within the BM Lin^−^ (Lin1 negative) population. * represents *p* < 0.05 (two-tailed Student’s *t*-test). Number of mice for each genotype used (*n* = 7) (data points for 2 *Rap1* WT and 3 *Rap1* KO mice were not included in Day 14 analyses because they died before the time point). **d** Cell cycle analysis with PI staining of the haematopoietic progenitor cells (KLS cells) and table summarizing the percentage of cells in G1, S, and G2M phases across the different genotypes. Number of mice for each genotype used (*n* = 3). **e** Annexin/PI staining of haematopoietic precursor (KLS cells) with/without 5 Gy irradiation and recovered for 24 h. Leftmost column depicts the gating of KLS cell populations. The rightmost panels depict the Annexin V (*X*-axis) and PI (*Y*-axis) analysis of KLS cells. Number of mice for each genotype used (*n* = 3). **f** Pictures of representative plates in the replatings are shown. Scale bar, 10 mm. **g** Colony-forming potential of total BM cells after irradiation. Colonies with more than 20 cells were scored from triplicate samples. Mean ± SEM of triplicate samples from *n* = 2/genotype is depicted. ***p < 0.001 (Mann–Whitney *U*-test).

To evaluate if Rap1 is involved in celluar stress response in vivo, we challenged littermate *Rap1* WT and KO mice with a lethal dose of (11 Gy) whole-body irradiation that causes DNA DSBs. *Rap1* KO mice were significantly more susceptible to irradiation and died within 4–8 days while WT controls survived longer (Fig. 1a). Loss of Rap1 also sensitized mice to DSB caused by chemotherapeutic agent 5-Fluorouracil (5-FU) (Supplementary Fig. 2A). Since BM is one of the most affected tissue compartments against DSB inducers, we next analyzed cellular changes in this compartment. We first checked the HSPC and myeloid progenitors in BM of mice subjected to a sublethal dose of irradiation (5 Gy) for defects in cellularity. An acute reduction in the KL and KSL cell populations was observed in *Rap1* KO mice compared to WT controls at 24 h and 2 weeks after irradiation (Fig. 1b, quantified in Fig. 1c). It is worth noting that there was no difference in the cell cycle profile of *Rap1* WT and *Rap1* KO mice (Fig. 1d). Annexin V/propidium iodide (PI)-staining apoptosis assay revealed a significant increase in cell death (upon 5 Gy treatment) in the *Rap1* KO mice compared to the *Rap1* WT mice (Fig. 1e). This suggests that loss of Rap1 rendered these cells unable to recover from radiation-induced damage due to enhanced apoptosis. To further test this notion, we analyzed the colony-forming potential of total BM cells from 5 Gy irradiated *Rap1* WT and KO mice. Rap1-deficient cells formed fewer colonies on methylcellulose plates compared to WT controls in the replating (Fig. 1f). This difference was further amplified when cells from the primary plate were replated (Fig. 1g). Taken together, these data suggest that the enhanced sensitivity of *Rap1* KO mice to genotoxic agents could be a consequence of BM failure in these mice due to the inability of Rap1 null HSPCs to proliferate and repopulate after irradiation.

### Rap1 null HSPCs accumulate more DNA damage after irradiation

**Fig. 2:**
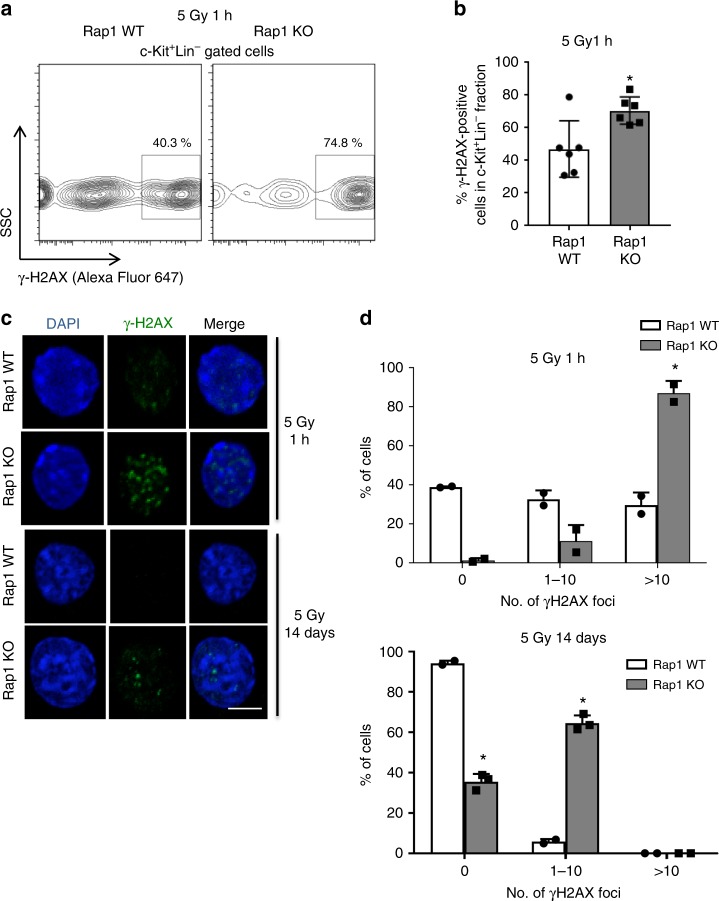
Loss of Rap1 leads to genomic instability. **a** Flow cytometric analysis of the γ-H2AX. Representative FACS plots of 30,000 gated cells on viable c-Kit^+^Lin^−^ (c-Kit positive, Lin1 negative) cells are shown. **b** Graphical representation of the results presented in **a** is shown. Mean ± SEM of the percentage of γ-H2AX-positive cells in the c-Kit^+^Lin^-^ fraction. Results represented are from two independent experiments (*n* = 6/genotype). * represents *p* < 0.05 (two-tailed Student’s *t*-test). **c** Representative images of the γ-H2AX staining in c-Kit^+^Lin^−^-derived cells using immunofluorescence staining. Scale bar, 10 μm. **d** Graphical representation of the γ-H2AX-foci numbers on 1 h and 14 days after irradiation, respectively. Results of γ-H2AX-foci scoring at 1 h (left) and 14 days (right) after 5 Gy irradiation are shown. Mean ± SD of duplicate experiments is depicted. *p < 0.05 (Mann–Whitney *U*-test).

To determine if the observed difference in irradiation sensitivity in these mice is due to defects in DNA damage repair, we performed intracellular flow cytometry staining and immunofluorescence analysis for phosphorylated H2AX (γ-H2AX), a marker for DNA damage, on HSPCs from irradiated mice. Within the cKit^+^lin^−^ cell population, there was a higher percentage of γ-H2AX-positive cells 1 h after irradiation in *Rap1* KO mice compared to the WT controls (Fig. 2a, b). Furthermore, while *Rap1* WT cells were able to resolve the DNA damage within 2 weeks post-irradiation, the γ-H2AX foci persisted in the cells from *Rap1* KO mice (Fig. 2c, d). Therefore, the increased sensitivity to irradiation observed in *Rap1* KO mice may be due, in part, to the inability of cells to heal the DNA damage effectively. Unresolved γ-H2AX foci in BM cells of *Rap1* KO mice were also observed in response to 5-FU injection (Supplementary Fig. 2B, C). To further demonstrate the increased sensitivity of the *Rap1* KO cells to genotoxic stress, comet assay was performed in *Rap1* WT and *Rap1* KO MEFs, and incresaed DNA fragmentation is observed in *Rap1* KO MEFs (Supplementary Fig. 2D, E). Taken together, these show that Rap1 loss impairs the ability of cells to resolve DNA damage.

### Rap1 loss accelerates tumorigenesis in Eµ-Myc mice

**Fig. 3:**
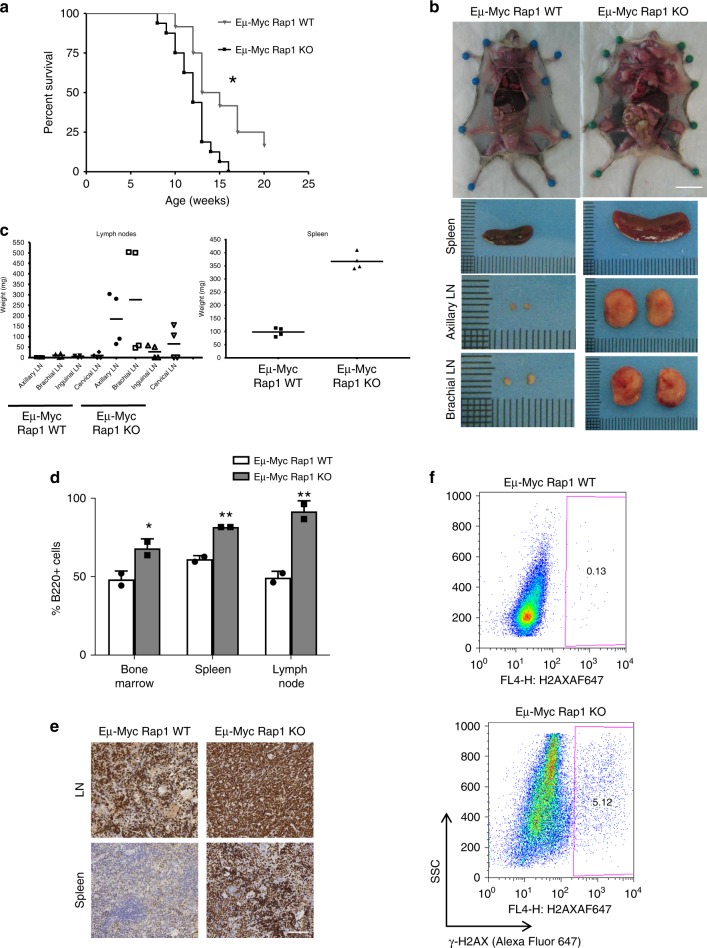
Myc-induced lymphomagenesis is accelerated in the absence of Rap1. **a** Kaplan–Meier survival curve of Eμ-Myc *Rap1* WT (*n* = 12) and KO (*n* = 16) mice. *p value <0.05. Samples were collected after 15 weeks. **b** Photographs of a representative pair of Eμ-Myc *Rap1* WT and KO mice with enlarged LN (scale bar, 2 cm). Photos shown below are representative of spleen and LN. Samples were collected after 15 weeks. **c** Weights of spleen and different LN from Eμ-Myc *Rap1* WT and KO mice are plotted (*n* = 4). **d** Percentage of B cells (B220^+^) derived from the bone marrow, spleen, and LN of Eμ-Myc *Rap1* WT and KO mice as determined by flow cytometry (*n* = 2/genotype). Mean ± SD of the percentage of B220^+^ (B220 positive) cells is depicted. Samples were collected after 15 weeks. *p < 0.05 (Mann–Whitney *U*-test). **e** Immunohistochemical Ki67 staining of Eμ-Myc *Rap1* WT and KO LN and spleen sections. Samples were collected after 15 weeks. Scale bar, 100 μm. **f** Intracellular γ-H2AX staining of B cells from spleen of Eμ-Myc *Rap1* WT and KO. Gated cells indicate percentage of γ-H2AX-positive cells. Samples were collected after 15 weeks.

Intact DDR signaling plays a crucial role in preventing oncogene-driven tumorigenesis^28,29^. To determine if Rap1-mediated regulation of DDR contributes to this process, we crossed *Rap1* KO mice with Eµ-Myc transgenic mice^30^, where B cell-specific overexpression of the Myc oncogene leads to lymphomas. Since overexpression of Myc has been shown to induce DNA damage^31–33^, accelerated tumorigenesis is expected with defects in DDR signaling. Indeed, in the absence of Rap1, lymphomagenesis was significantly accelerated, resulting in poorer overall survival and greatly increased tumor burden for Eμ-Myc mice in Rap1 null background compared to littermate Eμ-Myc *Rap1* WT controls (Fig. 3a). Eµ-Myc *Rap1* KO mice developed bigger tumors and had severely enlarged spleens and lymph nodes (LN) (Fig. 3b, c). Similarly, increased tumor sizes (and accelerated tumorigenesis) were observed in PyMT mice in Rap1 null background, compared to littermate PyMT controls (Supplementary Fig. 2F). B cell populations (B220^+^, where B220^+^ denotes B220-positive cells) in the BM, spleen, and LN of Eμ-Myc *Rap1* KO mice were significantly increased (Fig. 3d). In line with this, immunohistochemistry analysis of LN and spleen sections revealed higher levels of cell proliferation in Eµ-Myc *Rap1* KO mice, as evidenced by more intense Ki67 staining (Fig. 3e). Moreover, increased γ-H2AX staining was observed in splenic B cells in Eμ-Myc *Rap1* KO mice (Fig. 3f) suggesting that Rap1 delays disease progression in Myc-induced lymphomagenesis, possibly by regulating Myc-induced damage. Given that we see effects in Myc-driven and PyMT-driven cancers, it is likely that Rap1 plays a role in other types of stem cells.

### Rap1 is required for cell survival upon genotoxic challenge

**Fig. 4:**
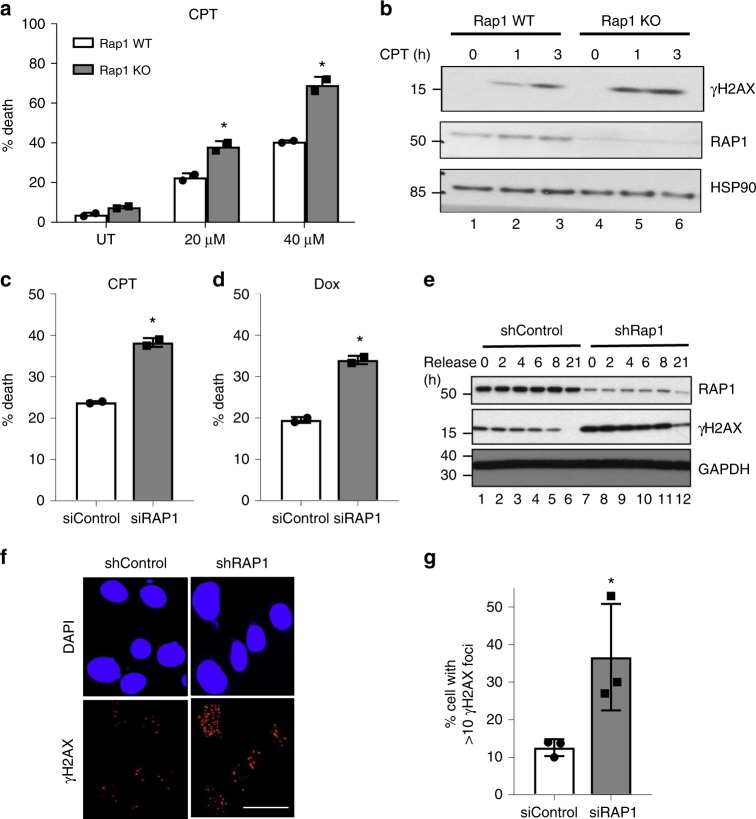
Rap1 promotes cell survival and DNA damage repair after chemotherapeutic drug treatment. **a** *Rap1* WT and KO MEFs were treated with various doses of CPT and analyzed for cell death after 48 h. Mean ± SD of duplicate experiments are presented. *p < 0.05 (two-tailed Student’s *t*-test). **b** Western blot analysis in *Rap1* KO and WT MEFs upon CPT treatment for indicated time points. **c**, **d** MCF7 cells were transfected with RAP1 or scrambled control siRNAs and cell survival was measured after 48 h treatment with CPT (60 μM) or Dox (1 μM) as indicated. Mean ± SD of the percentage cell death in duplicate experiments is depicted. *p < 0.05 (two-tailed Student’s *t*-test). **e** Western blot analysis in shControl and shRAP1-transfected MCF7 cells and released for different time points after CPT (10 μM, 3 h) treatment. **f** Immunofluorescence staining of γ-H2AX after CPT treatment (10 μM, 3 h) in RAP1 knockdown and control MCF7 cells (×60 magnification). Scale bar, 20 μm. **g** Quantification of **f**. Mean ± SEM of three separate fields is depicted. Each experiment was repeated three times and *p < 0.05 (two-tailed Student’s *t*-test).

Similar to irradiation and 5-FU accelerated death in *Rap1* KO mice, camptothecin (CPT) treatment also increased cell death in *Rap1* KO MEFs (*n* = 2) compared to WT MEFs (*n* = 2) and increased γ-H2AX accumulation (Fig. 4a, b). We extended these findings to human cells which are biochemically more tractable for further analysis. Similar results were obtained in human MCF7 cells in response to CPT, Doxorubicin (DOX) (Fig. 4c, d), Etoposide, and Mitomycin-C (Supplementary Fig. 2G, H) upon *RAP1* knockdown. As a positive control, DNA-PK knockdown also showed enhanced cell death upon CPT treatment (Supplementary Fig. 2I). DSBs are known to also activate NFκB signaling which protects cells from death^34,35^. Given that RAP1 activates NFκB signaling in response to cytokines^22^, we tested whether NFκB activation by genotoxic agents, which is initiated in the nucleus, is also regulated by RAP1. Knockdown of *RAP1* did not affect NFκB DNA binding upon treatment with Dox or CPT (Supplementary Fig. 2J), therefore, excluding the possibility that increased sensitivity to DSB inducers in the absence of RAP1 is due to decreased NFκB activation. This suggests that RAP1’s function in DNA damage could be independent of its role in transcription.

To test the level of damage upon *RAP1* knockdown in human cells, we compared the level of DNA damage in *RAP1* knockdown and control cells using γ-H2AX as a marker. Knockdown of *RAP1* using short-hairpin RNAs (shRNA) did not result in off-target effects on the shelterin complex or key NFκB signaling proteins (Supplementary Fig. 3A, B). Loss of RAP1 also caused increased accumulation of γ-H2AX upon CPT treatment in human cells (Fig. 4e). We also observed more γ-H2AX foci (Fig. 4f, g), colocalized with 53BP1 (Supplementary Fig. 3C), another marker of DNA damage. The quantification is presented in Supplementary Fig. 3D. Depletion of RAP1 does not induce significant TIFs (Supplementary Fig. 3E) as shown before^24^. To understand whether the damage foci preferentially reside on telomeres following CPT treatment, we costained γ-H2AX with TTAGGG-PNA probe and observed that damage foci were not specific to telomeres (Supplementary Fig. 3F). We confirmed that PNA probe specifically stained the telomeres (Supplementary Fig. 3F). These results show that RAP1-depleted cells accumulate more damage which is not limited to the telomeric regions, and that they concomitantly show more cell death.

### RAP1 binds to proteins involved in DNA damage repair

**Fig. 5:**
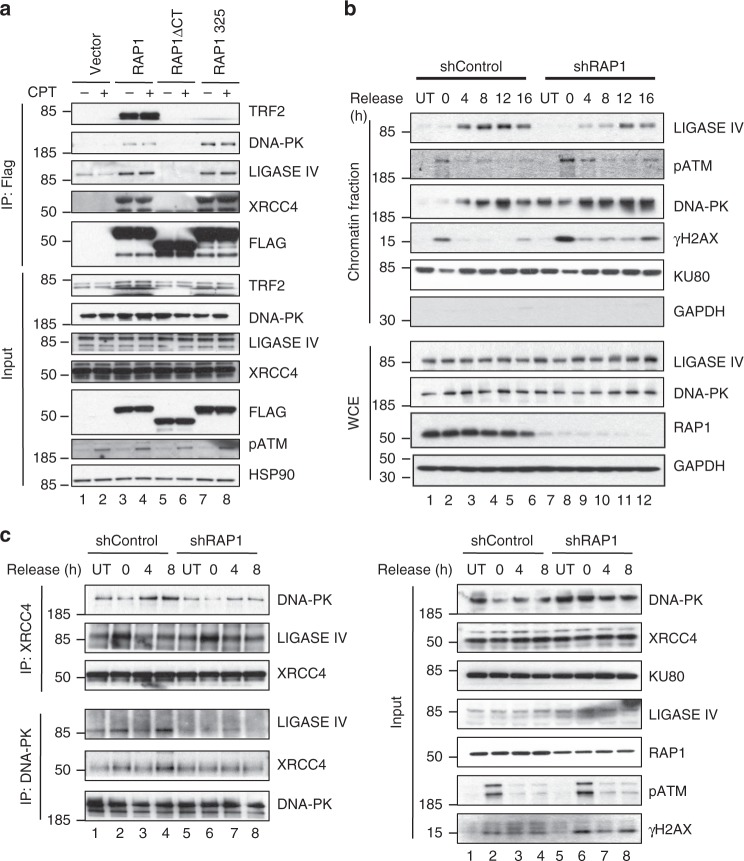
RAP1 mediates interaction between XRCC4/Lig IV and DNA-PK. **a** Flag-tagged RAP1 mutants (as indicated) were expressed in cells followed by IP using anti-Flag beads. The IP eluate was immunoblotted for the indicated proteins. Inputs are shown in bottom panel. **b** MCF7 cells were transfected with shcontrol or shRAP1, and left untreated (UT) or treated with CPT (10 μM) for 3 h (represented as 0 time point). Following this, cells were released for various time points and fractionated and blotted for indicated proteins. WCE indicated whole-cell extract **c** Co-immunoprecipitation of lysates from shRAP1 or shControl cells UT or treated with CPT (10 μM) for 3 h and released for the indicated times. Immunoblots demonstrate association of indicated proteins.

To identify possible RAP1 interactions with proteins that regulate DDR, we performed stable isotope labeling of amino acids in cell culture followed by mass spectrometry (SILAC-MS) using Flag-RAP1-expressing 293T cells (Supplementary Fig. 4A). Proteins with high SILAC ratios (>3) were then subjected to pathway analysis using GeneGO (MetaCore^TM^, version 6.11). A ratio-intensity plot of the RAP1-interacting proteins showed that RAP1 associates with several important members of the DDR pathway, including DNA-PK, Ku80, Ku70, Rad50, and MRE11 (Supplementary Fig. 4B). Indeed, among the RAP1-interacting proteome, the DDR pathway proteins were highly enriched (Supplementary Fig. 4C) (*p* < e-22). We further confirmed the interactions between endogenous RAP1 and DDR pathway members using size exclusion chromatography^22^ and endogenous RAP1 IP. We find that RAP1 interacts with many DDR proteins such as DNA-PK, DNA Ligase IV (Ligase IV), and ATM in untreated cells (Supplementary Fig. 4D). More importantly, the size of these complexes increased upon treatment with genotoxic drugs, Dox and CPT (compare lane numbers 1 vs. 7 and 13 in IP: RAP1 gel) (Supplementary Fig. 4D). This indicates that RAP1 may be a part of large functional complexes that regulate DDR upon genotoxic stress. Association of RAP1 with some DDR pathway members suggests that RAP1 may function as an adapter protein required for efficient signaling of the DDR pathway, which is reminiscent of its role in the canonical IKK signaling pathway^22^. To identify the domains of RAP1 required for binding to the various DDR proteins, we used Flag-tagged RAP1 mutants which have been previously reported^6^. Interestingly, RAP1 binds to different proteins via different domains (Supplementary Fig. 4E). RAP1 mutants lacking the CT or M/C domains were unable to bind to DNA-PK while TRF2 required the CT domain for binding. This suggests that while RAP1 may use different domains for binding different proteins, the CT domain appears to be the most important for interaction with multiple proteins. Since CT domain was required for binding with TRF2 as well as DDR proteins, we checked if RAP1-DDR protein interactions are TRF2 dependent. To this end, we made six amino acid linker scanning mutations in CT domain of RAP1 between residues 325 and 330 (named as RAP1–325). Consistent with previous findings that amino acid residues around 318 are necessary for TRF2 binding to human RAP1 (ref. ^36^), we observed that RAP1–325 was unable to bind to TRF2 (Fig. 5a). Importantly, RAP1–325 was able to bind to DDR proteins like DNA-PK, XRCC4, and Ligase IV (Fig. 5a), suggesting that its interactions with these DDR members are independent of TRF2.

### RAP1 mediates XRCC4/Ligase IV and DNA-PK interaction

To understand the role of RAP1 in DDR, we evaluated the kinetics of recruitment of these proteins on chromatin in cells treated with CPT and subsequently released for repair. Strikingly, the recruitment of Ligase IV to chromatin was impaired when RAP1 was depleted (Fig. 5b). Interestingly, association of DNA-PK and pATM with damaged chromatin was increased when RAP1 was depleted, possibly due to persistent unresolved breaks (Fig. 5b). This data suggested that RAP1 may be functioning as a platform that helps to recruit different complexes for efficient repair. To further characterize the function of RAP1, we analyzed the formation of DNA repair complexes prior to their association with chromatin by co-immunoprecipitation. While XRCC4 associated with DNA-PK, an association required for efficient repair in response to DNA damage, this interaction was markedly reduced in the absence of RAP1 (Fig. 5c). However, the association between Ligase IV and XRCC4 was unaffected by levels of RAP1. Further, a reverse IP demonstrated that DNA-PK interaction with Ligase IV/XRCC4 was markedly diminished in the absence of RAP1 (Fig. 5c). These experiments support the hypothesis that RAP1 interacts with DNA repair proteins XRCC4/Ligase IV and DNA-PK to promote DNA damage repair.

### Rap1 affects key NHEJ processes in vivo

**Fig. 6:**
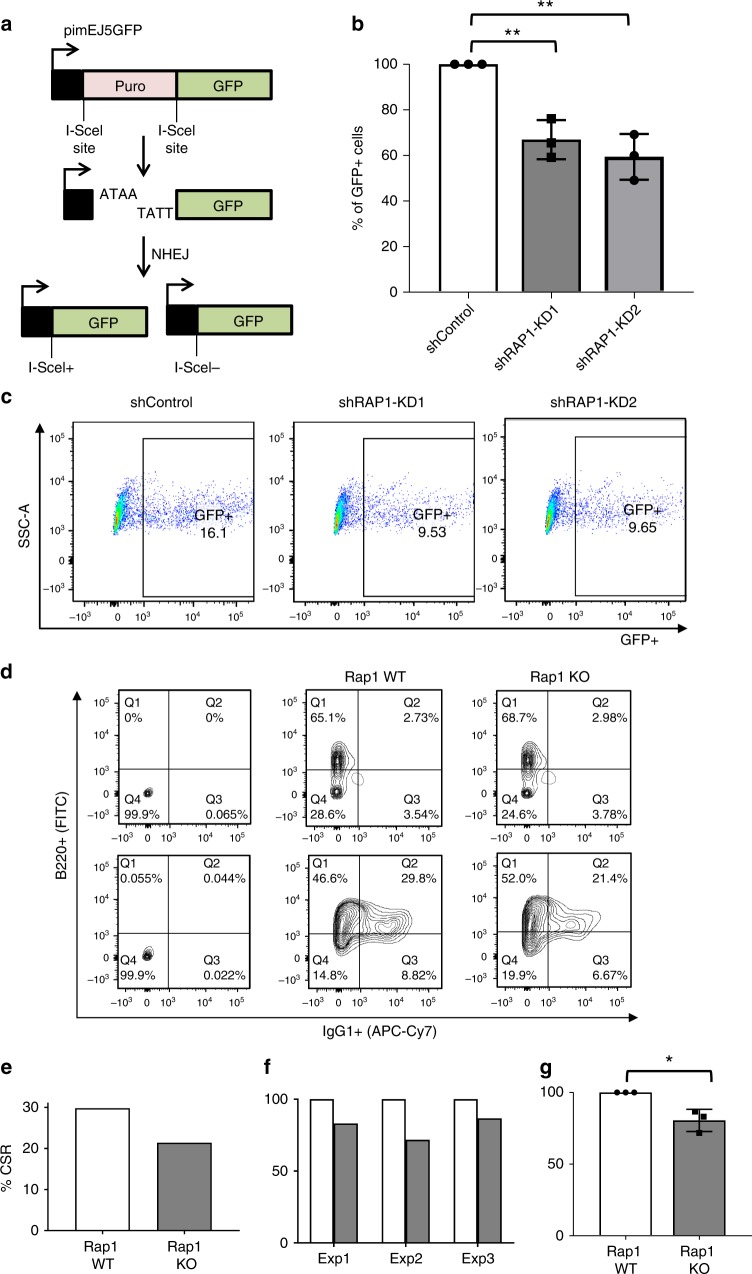
Rap1 regulates regulates NHEJ in vivo. **a** Schematic of cell-based repair assay (adapted from Bennardo et al.)^37^. **b** Average percentage of GFP-positive cells as a readout for NHEJ activity in RAP1 WT and KD cell lines from three independent experiments normalized to average percentage of GFP-positive cells in the WT cell line (Mean ± SEM) (**p < 0.01) (one-way ANOVA; Turkey’s multiple comparison test) (Scr = scrambled, KD1 = shRAP1 construct 1, KD2 = shRAP1 construct 2). **c** Representative FACs analysis of GFP-positive cells (corresponding from left to right: Scr, KD1, and KD2). **d** Representative FACs contour plots of B220 and IgG1 doubly stained B cells undergoing in vitro CSR via stimulation with LPS and IL4. Top panel represents B cells from the respective genotypes stained on D0 (day 0) of CSR; bottom panel represents B cells from the respective genotypes stained on D3 of CSR. Leftmost panel depicts unstained cell population. **e** Representative plot of % CSR in WT vs. *Rap1* KO B cells of a representative biological replicate. **f** Plot of CSR in WT vs. *Rap1* KO B cells, normalized to WT B cell CSR, over three independent biological experiments. **g** Mean ± SD of Averaged deficiency in CSR between WT vs. *Rap1* KO B cells across three independent biological replicates (*p < 0.05) (two-tailed Student’s *t*-test).

Given that our assays reveal a role for RAP1 in DNA damage repair, we next aimed to strengthen this claim by directly testing RAP1’s function in DDR using physiological in vivo assays. Consistent with the impaired recruitment of Ligase IV, the DSB repair activity in RAP1-depleted cells was lower compared to control cells. A cell-based double vector system previously described by Bennardo et al.^37^ was used to probe the efficiency of NHEJ in RAP1-depleted cells (Fig. 6a). Control and *RAP1* knockdown MCF7 cells were transiently transfected and the repair efficacy was measured using GFP as a readout. There was a statistically significant reduction in NHEJ repair activity in the RAP1 KD cells compared to the control cells (Fig. 6b, c), supporting the role for RAP1 as an adaptor protein in NHEJ DNA repair.

Next, we wanted to analyze the effect of Rap1 deficiency on B cell class switching, a physiologically relevant process where NHEJ repair plays an important role^38–40^. Splenic B cells isolated from WT and *Rap1* KO mice were stimulated with LPS and IL-4 cytokines to undergo IgG1 class switching. B cells isolated from *Rap1* KO mice displayed significantly reduced IgG1 class switching (representative FACs plots and quantification: Fig. 6d, e; percentage decrease normalized to WT over three experiments: Fig. 6f; averaged over three experiments: Fig. 6g). Together, these experiments provide in vivo evidence that Rap1 deficiency affects physiologically relevant processes where NHEJ mediated repair plays an important and non-redundant role. Taken together, this reinforces our hypothesis that despite not being a cannonical member of the NHEJ repair pathway, RAP1 enhances NHEJ activity by working as a potential adaptor in specific contexts.

### RAP1 levels predict chemotherapy response and survival

**Fig. 7:**
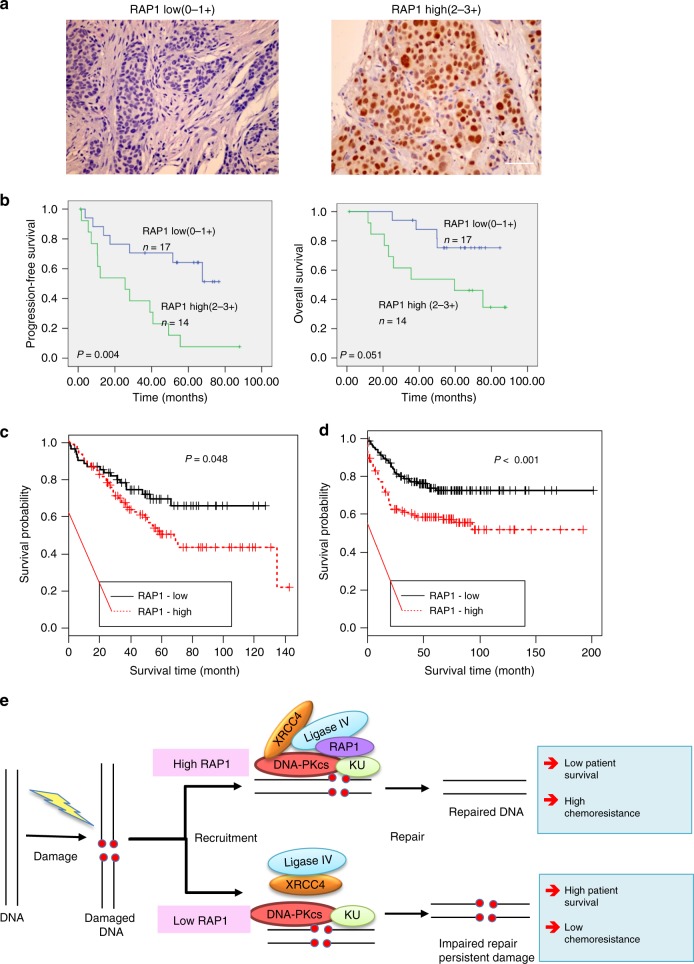
RAP1 levels correlate with response to chemotherapy and patient survival. **a** IHC staining of RAP1 in breast cancer patient tissues. Staining intensity was classified into four groups: 0 (negative), 1 (weak), 2 (moderate), and 3 (strong). Staining scores 0 and 1 were grouped as negative immunoreactivity whereas 2 and 3 were grouped as positive immunoreactivity. Scale bar, 50 μm. **b** Graphs showing correlation of progression free and overall survival with RAP1 levels (*n* = 31). **c**, **d** For survival data analysis of colorectal cancers, raw gene expression data with GEO accession numbers of GSE17538 (*n* = 232) and GSE39582 (*n* = 1193) were downloaded from the GEO database (http://www.ncbi.nlm.nih.gov/geo/). Normalization of the raw data was performed across all samples based on the Cross Correlation method^63^. Stratification of samples replied on the distribution of RAP1 (TERF2IP) expression intensities cross all samples. Ten percent below or over the RAP1 median expression value was used as the cutoff for selection of RAP1 lower or higher samples, respectively. The analysis of the survival data was based on the Kaplan–Meier method. **e** Model showing that RAP1 binds DDR complex proteins and regulates their formation and recruitment on chromatin.

To further investigate the relevance of our findings in human cancer patients, tissue microarray (TMA) blocks containing serial tumor cores from 100 breast cancer patients were constructed as described previously^41^. As a result of tissue loss during TMA construction and immunohistochemical processing, the following number of cases were available for evaluation: ER-negative patients, *n* = 17 and ER-positive patients, *n* = 31. We used ER-positive patient samples for IHC with RAP1. RAP1 staining on TMA blocks containing serial tumor cores were graded according to the intensity into four groups: 0 (negative), 1 (weak), 2 (moderate), and 3 (strong). Staining scores 0 and 1 were grouped as negative immunoreactivity whereas 2 and 3 were grouped as positive immunoreactivity (Fig. 7a). These patient samples were analyzed for correlation between RAP1 expression and patient survival. Importantly, patients with higher RAP1 levels responded poorly to chemotherapy as their tumors were resistant to death, and they were found to have reduced overall survival (OS) and progression-free survival (PFS) (Fig. 7b). Indeed, when cells are treated with CPT, the levels of RAP1 were found to be increased in nuclear fraction of cells (Supplementary Fig. 4F). To further strengthen the notion that RAP1 levels could be predictive of chemotherapy outcome, and to test the generality of our findings, we evaluated another chemotherapeutic regimen using CPT, which has been shown to be efficacious in colon cancer treatment. Indeed, survival analysis on two independent studies^42,43^ showed that reduced RAP1 levels predict better survival (Fig. 7c, d). Collectively, these results invoke a role for RAP1 in efficient recruitment of DNA repair complexes on chromatin upon DSB induction. A model based on our studies is presented (Fig. 7e). We suggest that RAP1 expression levels could be used as a prognostic tool for response to chemotherapy.

## Discussion

Here, using mouse models, human cancer cells, and data from phase II clinical trials, we suggest that mammalian Rap1 is an important regulator of genome stability in response to genotoxic and oncogenic signals, and it functions by controlling the recruitment of Ligase IV to the damaged chromatin due to its ability to serve as an adaptor. Our results do not exclude the possibility that Rap1 also functions in pathways that lead to repair, other than the one elucidated here, especially since it is clear that its interactome (Supplementary Fig 4A, B) is rich with DDR protiens. The covalent modifications of Rap1 which ensue upon specific kinds of damage and allow it to function effectively as an adaptor need further examination. Indeed Rap1 has context dependent functions as exemplified by the obesity phenotype which is seen only in Rap1 null females^23,24^.

Although mammalian Rap1 is one of the most conserved proteins, we have only just begun to understand its role, since unlike yeast Rap1, the mammalian Rap1 cannot bind DNA and since Rap1 null cells have no telomere defects. Extensive studies performed by the de Lange and Chang laboratories have shown that Rap1 is required for the prevention of aberrant Holliday junction resolution at telomeres by increasing T-loop formation, and that the loss of Rap1 results in aberrant homologous repair of the telomeres ^9,44^. This led us to hypothesize that there must be a reason for the conservation of this protein. Further, not only does its levels increase in cancers, additional mutations which lead to loss of its C terminus in cancers have recently been described. In this manuscript we provide evidence that mammalian Rap1 directly regulates DNA damage signaling, independent of its association with the telomeres or with its known partner TRF2. Several lines of evidence have previously hinted at the involvement of yeast and mammalian Rap1 in DDR. Firstly, RAP1 has been found to be phosphorylated in response to IR and UV in human cells^45^ and associated with various components of DDR machinery^7^. Secondly, RAP1 levels were upregulated upon DNA damage^46^. Also, yeast Rap1 was shown to have an important role in genome stability^47^. However, the precise contribution of mammalian Rap1 in this context was enigmatic. The participation of DDR and checkpoint activity in tumor suppression is highly appreciated in the context of oncogene-driven tumorigenesis^48^. The poorer survival of Eμ-Myc mice when Rap1 is null is much like the phenotype of this oncogene in ATM null background^49^, further increasing confidence in the notion that lack of Rap1 abrogates oncogene-induced DNA repair. Rap1 deficiency led to defects in DNA damage foci resolution, ultimately leading to cell death. This sensitivity to genotoxic stress is similar to that observed for the various members of the DNA damage pathway^50–52^ such as ATM, DNA-PK, Ligase IV, KU, and ATR. Enzyme-mediated repair of DSBs is a major mechanism of resistance to both ionizing radiation (IR) and drugs that cause DSBs. Thus, helping cells repair damaged DNA may be one of the important functions of RAP1 that leads to better cancer cell survival and worse patient surival. Importantly, this has been observed in breast cancer patients and colon cancer patients undergoing chemotherapy (Fig. 7).

The seemingly discordant data between accelerated death and lymphomagenesis in Eμ-myc-positive mice when Rap1 is null, but increased human patient survival when RAP1 is low need further explanation. Notwithstanding the differences between mice and humans, the major point of difference is that the human patient cohorts that are described were treated with chemotherapy (unlike the mice). Low levels of RAP1 lead to reduced repair of damaged DNA in tumor cells and this causes increased death of tumors which translates into better patient survival post chemotherapy. On the contrary, human tumors cells with high RAP1 repair more efficiently the DNA damage caused by chemotherapy drugs. Due to increased repair, RAP1-high human tumors survive better. This classical chemoresistance phenotype is well characterized in cancer patients. When tumor cells survive more, there is poorer patient survival (due to subsequent processes like metastasis). It is important to note that the increased expression of most DNA repair genes causes resistance to genotoxic compounds in human cancer. Adriamycin resistance in leukemic/breast cells is correlated with increased FANCD2 and FANCF expression^53^ as well as increased NFκB expression^54^. Similarly, since chemoresistant human tumors survive, proliferate, and metastasize more, RAP1-high patients survive less. This further reinforces our hypothesis that RAP1 has a non-telomeric function as an adaptor in the NHEJ pathway, away from the telomeres. The accelerated lymphomagenesis in mice also fits beautifully with the known role of DNA damage proteins in the process. Indeed, the loss of many key DNA damage repair proteins such as ATM^55^, MSH2 (ref. ^56^), and INK4a/ARF^57^ has been shown to accelerate Eμ-Myc lymphomagenesis, much like what is seen with Rap1 loss. Together these results confirm that Rap1 is a bone fide DNA damage repair protein.

Absence of RAP1 led to reduction in formation of a functional repair complex due to lack of association of XRCC4/Ligase IV with DNA-PK leading to impaired recruitment to damaged chromatin. This observation is important in the context of cancer or BM failure where efficiency of DDR is the key to cell survival^58^. This too is reminiscent of the effect of loss of DDR mediators such as ATM or the NHEJ complex proteins^50,51^. We further mapped the domains of RAP1 required for binding to different DDR members and found that the CT domain was important for most of the interactions (Supplementary Fig 4E). However, these interactions were independent of TRF2 since the RAP1–325 mutant which is unable to bind to TRF2 can efficiently bind to DNA-PK, Ligase IV, and XRCC4 proteins (Fig. 5a). Indeed, several human cancer patients have been found to harbor nonsense or deletion mutations right before CT domain of RAP1 (Supplementary Fig. 5)^59,60^, which could generate a RAP1 mutant which is unable to interact with these repair proteins and thus affect disease progression and response to chemotherapy. Taken together, our data uncover a previously uncharacterized aspect of RAP1 function in cellular response to DNA damage. This non-telomeric adaptor role of RAP1 could make RAP1 a good biomarker and therapeutic target in chemotherapeutics for human cancer.

## Methods

### Cells and reagents

*Rap1* WT and KO MEFs were derived by timed mating of *Rap1*^+/−^ (*Rap1* heterozygous) mice. Briefly, embryos were harvested at E13.5, internal organs removed, and fibroblasts cultured in Dulbecco’s modified eagle medium (DMEM) supplemented with 10% heat-inactivated fetal bovine serum (FBS), 2 mM l-glutamine, 2 mM sodium pyruvate, 2% β-mercaptoethanol, and 1× PSF (penicillin, streptomycin, and fungizone) at 37 °C with 10% CO_2_. MCF7 cells (ATCC® HTB-22™) and 293T (ATCC® CRL-3216™) were grown in DMEM; all media were supplemented with 10% FBS, 2 mM l-glutamine, 2 mM sodium pyruvate, and 1× PSF at 37 °C with 5% CO_2_. Antibodies against CHK2 (Cell Signaling Technology: #2662), pCHK2 (Cell Signaling Technology: #2661), ATM (Cell Signaling Technology: #2873 S), and KU80 (Cell Signaling Technology: #2180) were from Cell Signaling Technology. Rap1 (Santa Cruz: sc-53434 and sc-28197) was from Santa Cruz Biotechnology, TRF2 (Millipore: #05–521) and pATM (Millipore: #05–740) antibody were from Millipore. Ligase IV (Proteintech: #12695-1-AP) antibody was from Proteintech. XRCC4 (ab145) and DNA-PK (ab1832) antibodies were from Abcam and used at 1:2000 dilutions. All other antibodies were used at 1:1000 dilutions. Primers used in this study are listed in Supplementary Table 1.

### Transfection of plasmids and siRNA

Plasmids-overexpressing Flag-RAP1 was generated by cloning RAP1 ORF into pBOBi lentiviral plasmid. shRAP1 was generated by cloning target sequence into pLKO vector (sequence can be obtained upon request). RAP1 deletion mutants were a gift from Titia de Lange^6^. Linker scanning mutations in RAP1 CT domain were generated as described before^61^ and the mutants were cloned into Flag-tagged pBOBi. Lentiviruses were generated as described previously^22^. siRNA against RAP1 was obtained from Qiagen or Dharmacon. Control siRNA was from Qiagen. Cells were transfected using either Lipofectamine LTX or Lipofectamine RNAiMax for 48–72 h according to the manufacturer’s instructions.

### Cell death assay

Cells were plated 7 × 10^4^ per well of a 12-well plate in duplicates for 24 h. They were treated with Dox or CPT for 24 and 48 h. Thereafter, all cells in the supernatant as well as adherent cells were collected from each well and resuspended in an equal volume of phosphate-buffered saline (PBS). Equal volume of trypan blue dye was added to each sample. Total number of cells and dead cells were then counted using a hemocytometer. Cell death is presented as the percentage of dead cells compared to total cells in each well.

### Western blot analysis

Total protein was extracted with Totex buffer (20 mM Hepes at pH 7.9, 0.35 M NaCl, 20% glycerol, 1% NP-40, 1 mM MgCl_2_, 0.5 mM EDTA, 0.1 mM EGTA, 50 mM NaF, and 0.3 mM NaVO_3_) containing a mixture of protease inhibitors (Roche). Proteins were separated on NuPAGE Novex 4–12% Bis-Tris gels (Invitrogen) followed by immunoblotting with specific antibodies and detection using ECL Western blotting detection kit (Amersham Bioscience).

### Co-immunoprecipitation

Cells were washed with ice-cold PBS and then lysed in IP buffer (10 mM Tris at pH 8, 170 mM NaCl, 0.5% NP-40, and protease inhibitors) for 30 min on ice. Cell lysates were removed by centrifugation and the supernatants were incubated with Flag M2 antibody beads (sigma) or other antibodies as indicated overnight at 4 °C. Flag beads were washed four times with 1 ml of wash buffer (containing 200 mM Tris at pH 8.0, 100 mM NaCl, and 0.5% NP-40). For other antibodies, protein G sepharose beads were added for 2 h, rotated at 4 °C, and washed as flag beads. Bound proteins were eluted with 3X-Flag peptide or SDS sample buffer and subjected to western blot analysis.

### Cellular fractionation

The cells were harvested in ice-cold PBS and resuspended in hypotonic lysis buffer (10 mM HEPES pH 7.9, 1.5 mM MgCl_2_, 10 mM KCl, protease, and phosphatase inhibitors) and incubated on ice for 4 min. They were then spun down for 3 min at 1902 × *g* and the cytoplasmic fraction aspirated to separate tubes. The pellet was washed with hypotonic buffer once. The soluble nuclear fraction was then lysed in IP buffer on ice for 10 min. The lysate was clarified by centrifugation at 9391 × *g* for 10 min at 4 °C. For chromatin-bound fraction, pellet was resuspended in IP buffer and sonicated in Bioruptor with 30 s on/off cycle for 7 min. Clarified extract was used as chromatin fraction. Fractions were then analyzed using western blotting.

### NHEJ DSB repair assay

DSB repair assay was performed as described by Bennardo and colleagues. Using MCF7 RAP1 knockdown cell lines (KD1 and KD2) and control cell line (Scr), 1 × 10^6^ cells were seeded into six-well plates. The cells were transfected with pCBASceI and pimEJ5GFP using X-tremeGENE™ 9 DNA Transfection Reagent (6365779001) the following day with the manufacturer’s protocol. The NHEJ DSB assay was performed 3 days after the transfection. The cells were harvested and washed with PBS, and resuspended in 10% FBS in PBS. The cells were put through the flow cytometric analyzer and 10,000 events were recorded for each sample. The results were analyzed using FlowJo.

### Comet assay

Comet assay was performed using CometAssay® Kit (25 × 2 well slides) from Trevigen (4250–050-K) following the manufacturer’s protocol with slight modifications. *Rap1* WT and KO MEFs were seeded in six-well plates (0.2 × 10^6^ cells per well). The cells were treated with 10 μM of CPT for 3 h before harvesting. The cell pellets were resuspended in 200 μL of cold PBS. One hundred and twenty microliters of 0.75% agarose was added to 10 μL of cells (0.01 × 10^6^ cells) and pipetted onto the microscopic slides. The gel was allowed to set at 4 °C for 30 min. The remaining steps were performed following the manufacturer’s protocol and the slides were imaged using the Zeiss LSM 800 and quantitated using ImageJ.

### Mice

*Rap1* KO mice on a mixed background were generated as described in the text. Briefly, *Rap1* conditional knockout mice were crossed to β-actin-Cre transgenic mice to generate whole-body *Rap1* knockout mice (KO, Rap1^−/−^). These mice were subsequently backcrossed six to seven times with C57BL6 mice to generate at least a 99.2–99.6% contribution of C57BL6, and used for the gamma irradiation, 5-FU and MEF experiments. These *Rap1*^−/−^ mice were also crossed to Eμ-Myc transgenic mice on a C57BL6 background^30^ to generate Eμ-Myc *Rap1*^+/−^ mice. Eμ-Myc *Rap1*^+/−^ and *Rap1*^+/−^ F1 mice were intercrossed to produce Eμ-Myc *Rap1*^+/+^, Eμ-Myc *Rap1*^+/−^, Eμ-Myc *Rap1*^−/−^, *Rap1*^+/+^, *Rap1*^+/−^, and *Rap1*^−/−^ offsprings. Eμ-Myc-positive littermates were monitored over the course of their disease and used for subsequent experiments. *Rap1* KO PyMT mice were generated by crossing *Rap1*^−/−^ mice with MMTV-PyMT mice (Jackson Laboratory). Animal experiments were approved by the Agency for Science, Technology and Research’s Biological Resource Centre Institutional Animal Care and Use Committee (*Rap1*^+/+^ denotes *Rap1* wild-type mice, *Rap1*^+/−^ denotes *Rap1* heterozygous mice, *Rap1*^−/−^ denotes *Rap1* knockout mice.)

### Class switch recombination assays

*Rap1*^−/−^ mice have been backcrossed for at least nine generations with C57BL6 mice to generate 99.9% contribution of C57BL6, before they were used for the class switch recombination assays. Briefly, splenic B cells were purified from the spleens of WT or *Rap1* KO mice using anti-CD43 magnetic beads. The B cells were stimulated for 72 h with 10 μg/ml LPS (Sigma) and 10 ng/ml IL-4 (R&D Systems) in RPMI media to induce IgG1 class switch recombination. B cells were harvested at the stipulated time points, washed with PBS, and stained with anti-B220 (BD Biosciences, 553092) and anti-IgG1 (BD Biosciences, 553441) antibodies and analyzed via flow cytometry.

### Analysis of peripheral blood cells

Peripheral blood (PB) was collected by retro-orbital bleeding and complete blood counts were performed using an automated hemocytometer, Hemavet (Drew Scientific).

### Isolation of BM cells

BM cells were flushed from the long bones (tibias and femurs) with minimum essential medium alpha medium (α-MEM) (Gibco) supplemented with 10% heat-inactivated FBS (Gibco). Cells were filtered through nylon filter (35 μm) to obtain a single-cell suspension.

### Flow cytometric analysis and cell sorting

For flow cytometric analysis, BM cells were preincubated with mouse serum for 15 min on ice before staining. The antibody reaction was carried out in the mouse serum for additional 20 min on ice. After addition of 0.5 μg/ml of Hoechst 33258 pentahydrate to exclude dead cells, analysis was performed using a LSR II flow cytometer (BD Biosciences) and analyzed using the FlowJo software (Tree Star). Sorting of KSL cells were performed using FACSAria (BD Bioscience).

For analysis and sorting of myeloid progenitors and HSPCs, cells were stained with antibodies against c-Kit (PE-Cy7), Sca-1 (APC), and lineage markers (PE) including CD3, CD4, CD8, B220, Gr-1, Mac-1, Ter117, and IL7Rα. Mature cells were identified as follows: myeloid, Mac1^+^ (Mac1 positive) and Gr1^+^; B cells, B220^+^ CD19^+^; erythroid cells, Ter119^+^; T cells CD4^+^ and CD8+. Antibodies were purchased from e-Bioscience and BD Pharmingen (San Diego).

For intracellular γ-H2AX staining, KL-sorted cells were first incubated in fixable viability dye-eFluor 780 (eBioscience) before fixation in 1% PFA on ice. The cells were then permeabilized with 0.25% Triton-X in 1% bovine serum albumin (BSA)-PBS on ice. Subsequently, cells were incubated with γ-H2AX antibody (Cell Signaling Technology) in 10% goat serum in 1% BSA-PBS and finally incubated with secondary antibody conjugated with Alexa Fluor 647.

### Cell cycle and apoptosis assay

For Annexin V apoptosis assay, stained cells were suspended in final buffer containing Annexin V antibody and Hoechst 33258 dye. All antibodies were purchased from BD Pharmingen (San Diego, CA, USA). For cell cycle analysis, cKIT-positive cells were sorted on a Becton Dickinson FACSAria II cell sorter (BD Biosciences), fixed in ice-cold 70% ethanol, and then treated with 1 mg/ml of RNAse A and stained with 500 μg/ml PI staining solution.

### Flow cytometric analysis of lymphoid development

Hematopoietic stem and progenitor cell and lineage-positive cell profiling was perform on BM, spleen, and thymus of mice from each genotype. Antibodies (clones) used: CD3 (145-2C11) [BD: #553064], CD4 (H129.19) [BD: #553653], CD8 (53-6.7) [BD: #553033], Gr-1 (RB6-8C5) [BD: #553128], B220 (RA3-6B2) [BD: #553090], Ter119 (Ter119) [BD: #553673], Mac1 (M1/70) [BD: #553311], IL7Rα (A7R34) [eBiosceinces: #12-1271-82], cKit (2B8) [BD: #558163], Sca-1 (E13-161.7) [eBiosceinces: #17-5981-83], CD25 (7D4) [eBiosciences: 13-0252-82], CD44 (IM7) [BD: 559250], CD43 (S7) [BD: 561856], HSA (M1/69) [BD: #553262] and BP-1 (6C3) [BD: #553159], CD4 (GK1.5) [BD: #561830], CD8 (7D4) [eBiosciences: #12-0081-82], B220 (RA3-6B2) [BD: #561880].

Flow cytometric gating strategy is described in Supplementary Fig. 6.

### Immunofluorescence staining

Sorted cells were collected onto glass slide using Cytospin4 cytocentrifuge (Fisher Scientific) and fixed using 4% PFA before permeabilized with 0.3% Triton X-PBS. All wash steps were carried out using 0.1% NP-40 in PBS (washing buffer). Slides were blocked overnight at 4 °C with 10% goat serum in washing buffer. Staining was carried out by incubating cells with anti-γ-H2AX antibody (Millipore: 05–636) and anti-53BP1 antibody (Cell Signaling Technology: #4937 S) further incubated with Alexa Fluor 488 and 647-conjugated secondary antibodies. Slides were then visualized on the A1 Confocal microscope system (Nikon). For γ-H2AX-TTAGGG-PNA probe co-staining, cells were fixed in 4% formaldehyde for 15 min, permeabilized with 0.2% (v/v) Triton-X at 4.0 °C for 10 min, and blocked with 0.5% BSA for 30 min. Primary antibody incubation with anti-γ-H2AX for 1 h was followed by three washes before incubation with secondary anti-rabbit antibody. Slides were then fixed in 4% formaldehyde to cross-fix antibodies before hybridization with telomeric PNA. For more details, please refer to Yasaei et al.^62^.

### CFU-C assay

In all, 50,000 sorted cKit^+^Lin^−^ cells were seeded in 35-mm dishes in either duplicates or triplicates in Methocult M3231 methylcellulose medium (StemCell Tec., Vancouver, BC, Canada) containing 1% antibiotic–antimycotic supplemented with 10 ng/ml recombinant murine IL-3, 10 ng/ml SCF, 10 ng/ml G-CSF, and 10 ng/ml EPO. Cells were incubated at 37 °C, 5% CO_2_. Colony formation was scored with an inverted microscope after 7 days of culture. Colonies consisting of more than 20 cells were scored. For serial replating assays, colonies were harvested in cold PBS, centrifuged at 440 × *g* for 5 min to collect cell pellet and colony assay was repeated using 50,000 cells for the next round.

### γ-Irradiation, 5-FU treatment, and Kaplan–Meier curve

*Rap1* WT, KO, and Het age-matched mouse cohorts (8–12 weeks old males, *n* = 5) were irradiated with 11 Gy and survival was monitored twice daily. 5-FU was dissolved in water and 150 mg/kg was injected intraperitoneally and mice were monitored for death. Readings were plotted in prism software and the Kaplan–Meier survival curve was plotted.

### Breast cancer patient samples

Patients Study Cohort: TMA blocks containing serial tumor cores from the 100 patients were constructed where four core biopsies from each patient were obtained: at baseline, 3 weeks after the first and second cycles of chemotherapy, and upon completion of six cycles of chemotherapy or at study withdrawal^41^. The cases were given randomized alternating sequences of doxorubicin (A) and docetaxel (T), starting either with doxorubicin 75 mg/m^2^ or docetaxel 75 mg/m^2^ every 3 weeks for six cycles. PFS and OS were defined as the time between the date of randomization and the first documented evidence of progression, and the time between the date of randomization and death, respectively.

### Scoring of RAP1 expression

The nuclear immunostaining of RAP1 was graded according to the intensity of positive staining in cancer cells. Staining intensity was classified into 4 groups: 0 (negative), 1 (weak), 2 (moderate), and 3 (strong). Staining scores 0 and 1 were grouped as negative immunoreactivity whereas 2 and 3 were grouped as positive immunoreactivity.

### Statistical analysis

Univariate and multivariate Cox proportional hazards models were carried out with PFS or OS as the end point. Survival analysis was conducted using Kaplan–Meier and the log-rank test was employed to determine the difference. All statistical analysis were performed using the SPSS package (version 18.0 for Windows; SPSS Inc., USA) with significance set at the 5% level.

### Colorectal cancer patient samples

Raw gene expression data with GEO accession numbers of GSE17538 (ref. ^42^) and GSE39582 (ref. ^43^) were downloaded from the GEO database (http://www.ncbi.nlm.nih.gov/geo/). In GSE39582 a total of 1193 colorectal cases were studied, and among Stage II–III cases, 418 cases received adjuvant chemotherapy of fluorouracil and folinic acid. In GSE17538 a total of 232 cases were studied.

### Gel filtration

Briefly, cells were harvested and resuspended in gel filtration buffer (20 mM Tris pH 7.5, 100 mM NaCl, 10 mM b-glycerol, 1 mM Benzamidine, 25 mM NaF, 1 mM DTT, 0.33 mM NaVO_4_) supplemented with protease and phosphatase inhibitors. Cells were sonicated and centrifuged for 15 min at 18407 × *g*. The supernatant was collected and filtered through at 0.22 μm filter before quantitation. The sample was then transferred into a 5 ml syringe for loading into the gel filtration system. For more details, please refer to Wu et al^34^.

### Reporting Summary

Further information on research design is available in the Nature Research Reporting Summary linked to this article.

## Supplementary information

Supplementary Information

Reporting Summary

## Data Availability

All data needed for interpretation of the results are presented in this paper; original source of big data sets are quoted in the Methods section. Source data underlying Figs. 1A, 1C, 1G, 2B, 2D, 3A, 3C, 3D, 4A-E, 4G, 5A–C, 6B, 6E–G, 7B, and Supplementary figure 1B, 1E, 1F, 2A, 2C, 2D, 2E, S2G–I, S3A–B, S3D, S4B–F have been made available.

## Source data

Source Data

## Supplementary information

**Supplementary information** is avaliable for this paper at 10.1038/s41467-019-13082-9.
