# Supplementary material for: Rap1 regulates hematopoietic stem cell survival and affects oncogenesis and response to chemotherapy
Source: Nat Commun. 2019 Dec 13;10:5349. doi: 10.1038/s41467-019-13082-9 (PMC6911077; doi:10.1038/s41467-019-13082-9)
Supplement: Supplementary file 1 — Supplementary Information [file 41467_2019_13082_MOESM1_ESM.pdf]

Rap1 regulates hematopoietic stem cell survival and affects oncogenesis and response to chemotherapy

Khattar et.al. 2019

Supplementary Information

Supplementary Figure 1

Supplementary Figure 2

Supplementary Figure 3

Supplementary Figure 4

Supplementary Figure 5

Supplementary Figure 6

Supplementary Table 1

Supplementary Figure 1

A

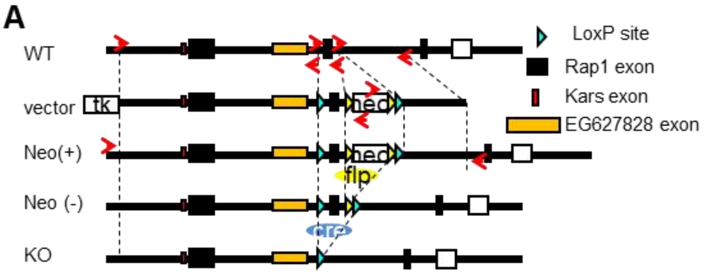

B

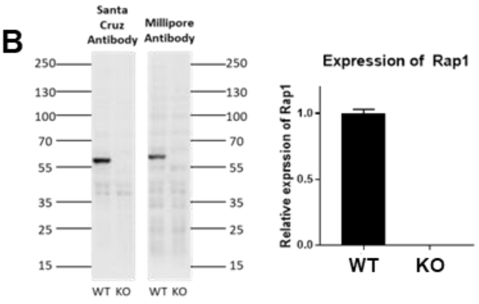

C

Parameters	Rap1 WT	Rap1 KO	p-value
White blood cell (x 10^3/μL)	7.88 ±0.72	7.36 ±0.68	n.s.
Hemoglobin (g/dL)	14.36 ±0.55	14.90 ±0.83	n.s.
Platelet (x 10^3/μL)	1104.6 ±73.67	894.2 ±58.40	n.s.

D

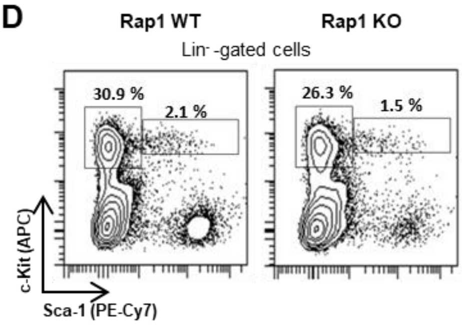

E

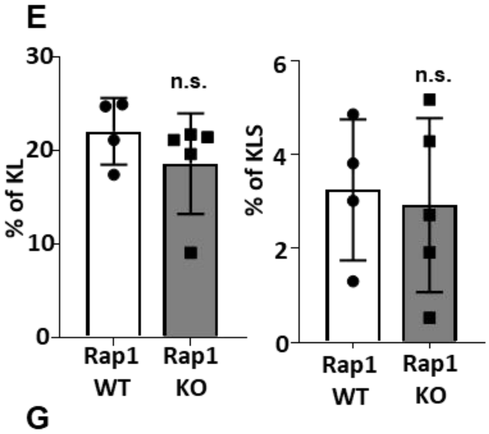

F

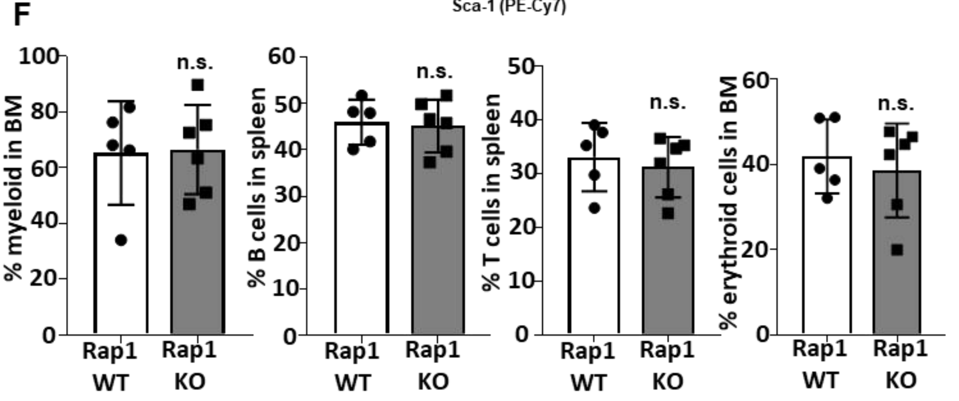

G

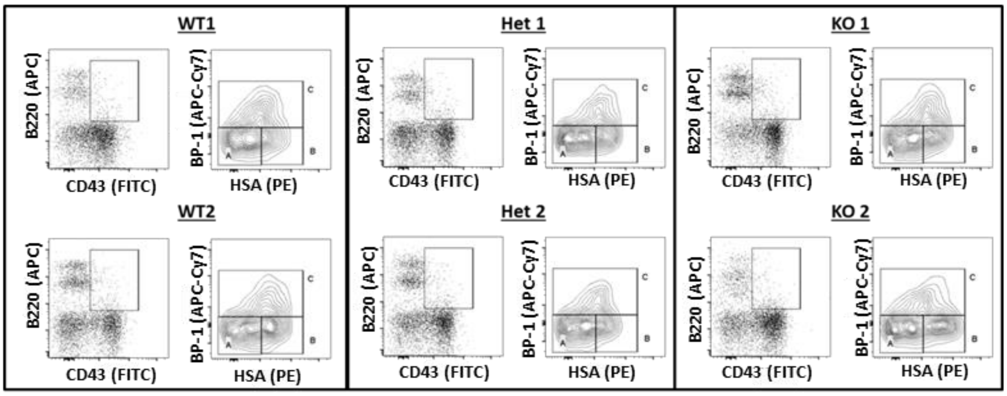

	Quadrants (%)		
Rap1	A	B	C
WT 1	46.0	17.1	32.8
WT 2	40.5	26.6	29.2
Het 1	46.6	22.6	27.7
Het 2	51.0	20.2	25.6
KO 1	43.4	23.5	29.3
KO 2	39.8	29.5	26.7

H

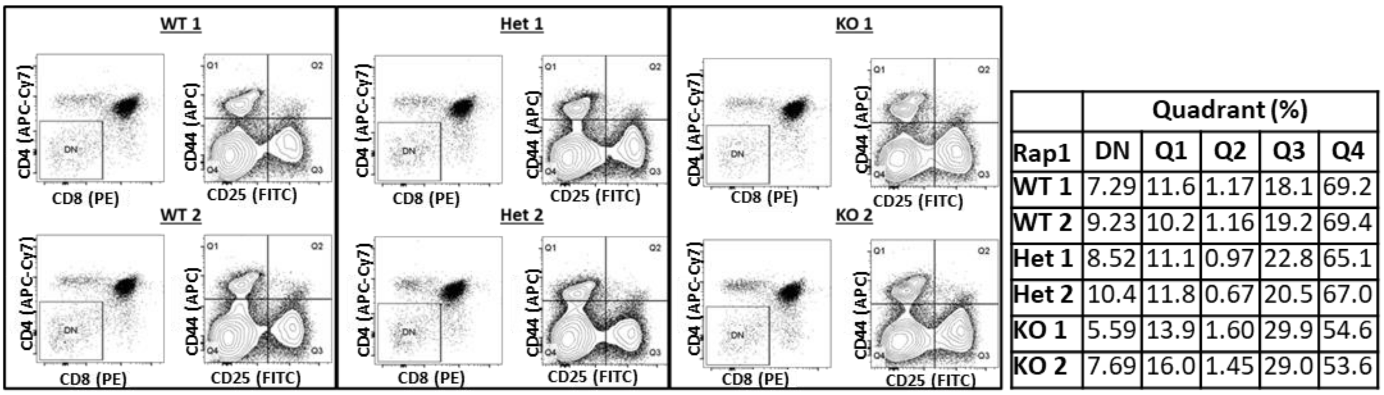

	Quadrant (%)				
Rap1	DN	Q1	Q2	Q3	Q4
WT 1	7.29	11.6	1.17	18.1	69.2
WT 2	9.23	10.2	1.16	19.2	69.4
Het 1	8.52	11.1	0.97	22.8	65.1
Het 2	10.4	11.8	0.67	20.5	67.0
KO 1	5.59	13.9	1.60	29.9	54.6
KO 2	7.69	16.0	1.45	29.0	53.6

Supplementary Fig. 1. Rap1 KO mice do not display gross hematopoietic abnormalities under normal unstressed conditions.

(A) Schematic describing the Rap1 locus in mice and the targeting construct. Boxed regions as per legend. Yellow arrowheads denote *Frt* sites for removing Neomycin; Blue arrowheards denote *LoxP* sites for removing Rap1 exon 2. Upon crossing with Cre-recombinase mice, the LoxP site recombination results in the inactive Rap1 allele as shown. (B) Western blot and qPCR of MEFs derived from Rap1 WT and KO mice. Error bar represent S.E.M. of technical triplicates. (C) Complete blood count analysis of peripheral blood. n.s.: no significant difference (2-tailed Student's t-test). (D) Flow cytometric analysis of HSPC compartment in BM of 6 to 8 weeks old mice. Representative FACS plots of 200,000 cells gated on viable Lin^- cells are shown. (E) Graphical representation of the results presented in D. Mean ± S.E.M of the percentage of KSL (right) and KL (left) within the BM Lin^- population (n = 4 to 5/genotype). (F) Graphical representation of the frequency of the cells of indicated lineage in the BM or spleen in 6 to 8 weeks old mice. Mean ± S.E.M from at least 2 independent experiments are shown in the graphs (n = 5 to 6/genotype). (G) FACs analysis of the B cell compartment in WT, Het and KO Rap1 mice. Left plots for each genotype depict the gating for B220 and CD43 double positive population. Right plots for each genotype depict the gating for the various pro B-cell populations (Fr.A, Fr.B and Fr.C) based on HSA/CD24 and BP-1 stainings. Table: percentage of cells in the respective quadrants. A represents Hardy Fr.A, B represent Hardy Fr.B and C represents Hardy Fr.C of the pro-B cell population (n=2 / genotype). (H) FACs analysis of the T cell compartment in WT, Het and KO Rap1 mice. Left plots for each genotype depict the gating for CD4 (Y-axis) and CD8 (X-axis) double negative (DN) population. Rightmost plots for each genotype depict the gating and the various T-cell population of cells based on CD25 (X-axis) and CD44 (Y-axis). Table: percentage of cells in the respective quadrants. Q1 represents DN1, Q2 represents DN2, Q3 represents DN3 and Q4 represents DN4 subpopulations of the DN population (n=2 / genotype).

Supplementary Figure 2

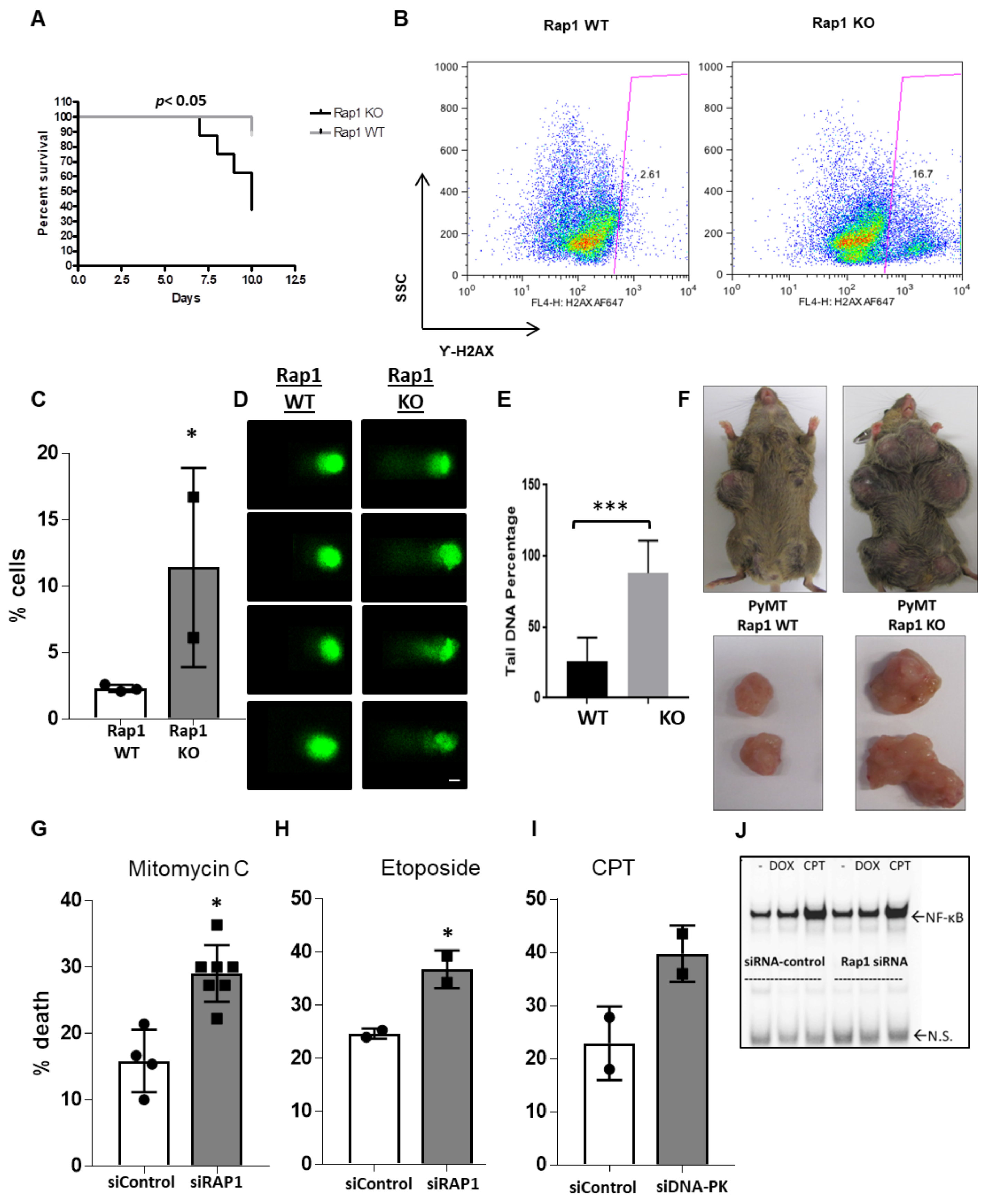

Supplementary Fig. 2. Rap1 depletion sensitizes cells to genotoxic stress. (A) Survival curve for Rap1 WT and KO mice treated with 5-FU. (B) Representative intracellular γ-H2AX staining 2-weeks after 5-FU treatment. (C) Quantification of (B). * represents $p < 0.05$ (2-tailed Student's t-test). Error bars represent S.E.M. of triplicate experiments (Data point for 1 *Rap1* knockout mouse was not included in the analyses because it died during the experiment). (D) Representative images of comet assay performed using Rap1

WT and KO MEFs. Scale bar, 100µm. (E) Quantification of (D). Error bars represent S.D. of five separate fields. * represents $p < 0.05$ (2-tailed Student's t-test). (F) Representative images of Rap1 WT-PYMT and Rap1 KO-PYMT mice (top panel) and the tumors extracted from the respective mice (bottom panel). (G) - (I) MCF7 cells were transfected with RAP1, DNA-PK or scrambled control siRNAs and cell survival was measured after 48h treatment with mitomycin C (10µg/ml) Etoposide (50µM) or CPT (60µM). Error bars represent S.E.M. of at least duplicate experiments. * represents $p < 0.05$ (2-tailed Student's t-test). (J) Western blot analysis in shControl and shRAP1 transfected MCF7 cells and released for different time points after CPT (10µM, 3h) treatment.

Supplementary Figure 3

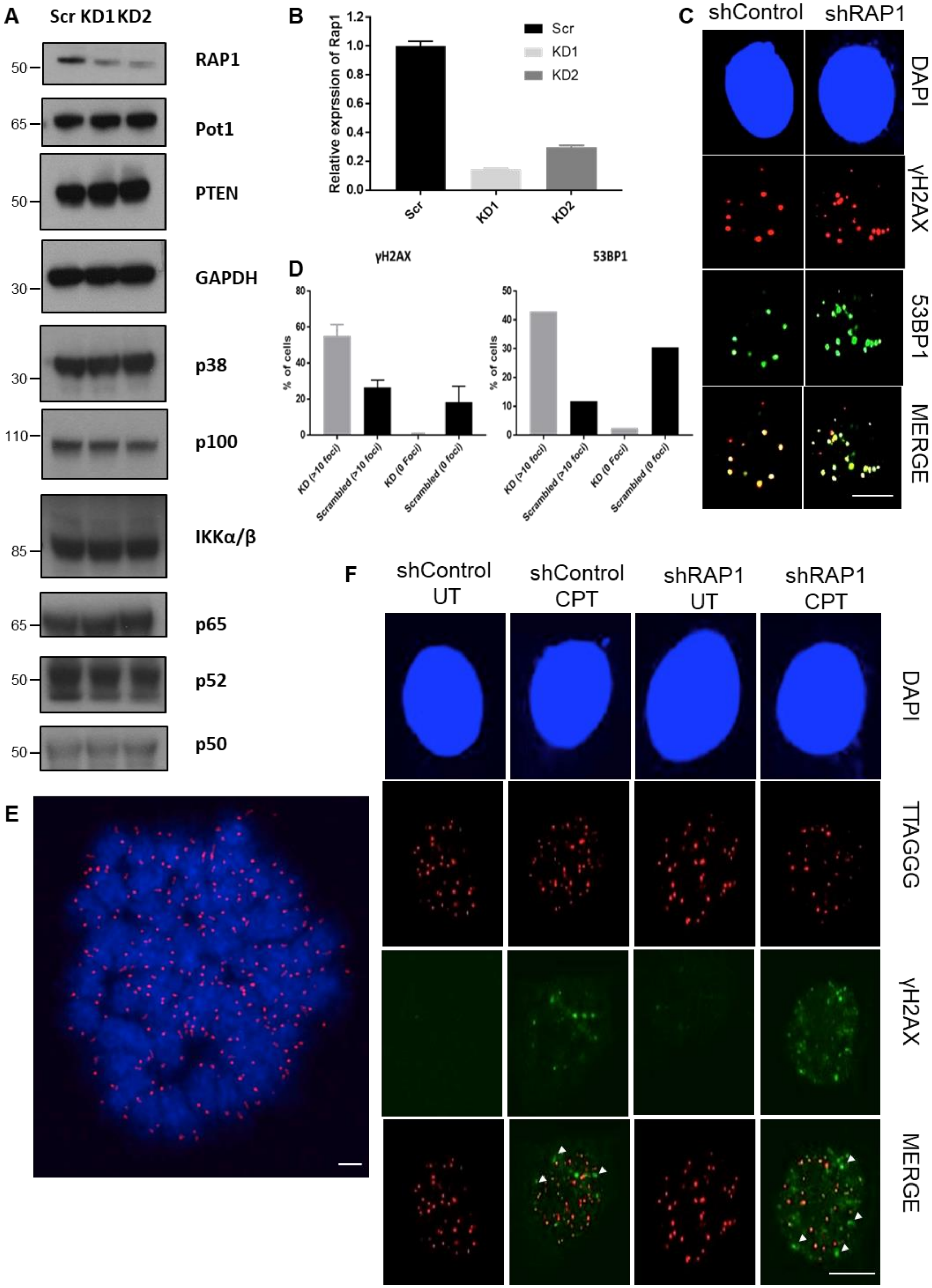

Supplementary Fig. 3. Increased γ-H2AX foci upon Rap1 depletion do not specifically localize to telomeres

(A) Western blot of other shelterin complex/NF-κB pathway proteins in the Rap1 knockdown cell lines using shRNA knockdown in MCF7 cell line (Scr=scrambled, KD1=shRAP1 construct 1, KD2=shRAP1 construct 2). (B) qPCR of RAP1 levels in the scrambled and knockdown MCF7 cells. Error bar represent S.E.M. of technical triplicates. * represents $p < 0.05$ (2-tailed Student's t-test). (C) Immunofluorescence co-staining of γ-H2AX and 53BP1 after CPT treatment (3h) in *RAP1* knockdown and control MCF7 cells (60x magnification). Scale bar, 5μm. (D) Quantification of (C). Error bar represent S.D. of ten separate fields from each of the triplicate experiments. * represents $p < 0.05$ (2-tailed Student's t-test). (E) Telomere Fluorescence In Situ Hybridization (TELO-FISH) staining in metaphase spreads of MCF7 cells (100x magnification). Scale bar, 2μm. (F) Immunofluorescence co-staining of γ-H2AX and TTAGGG-PNA probe. Arrows denote some γ-H2AX foci not colocalizing with telomeres. Scale bar, 5μm.

Supplementary Figure 4

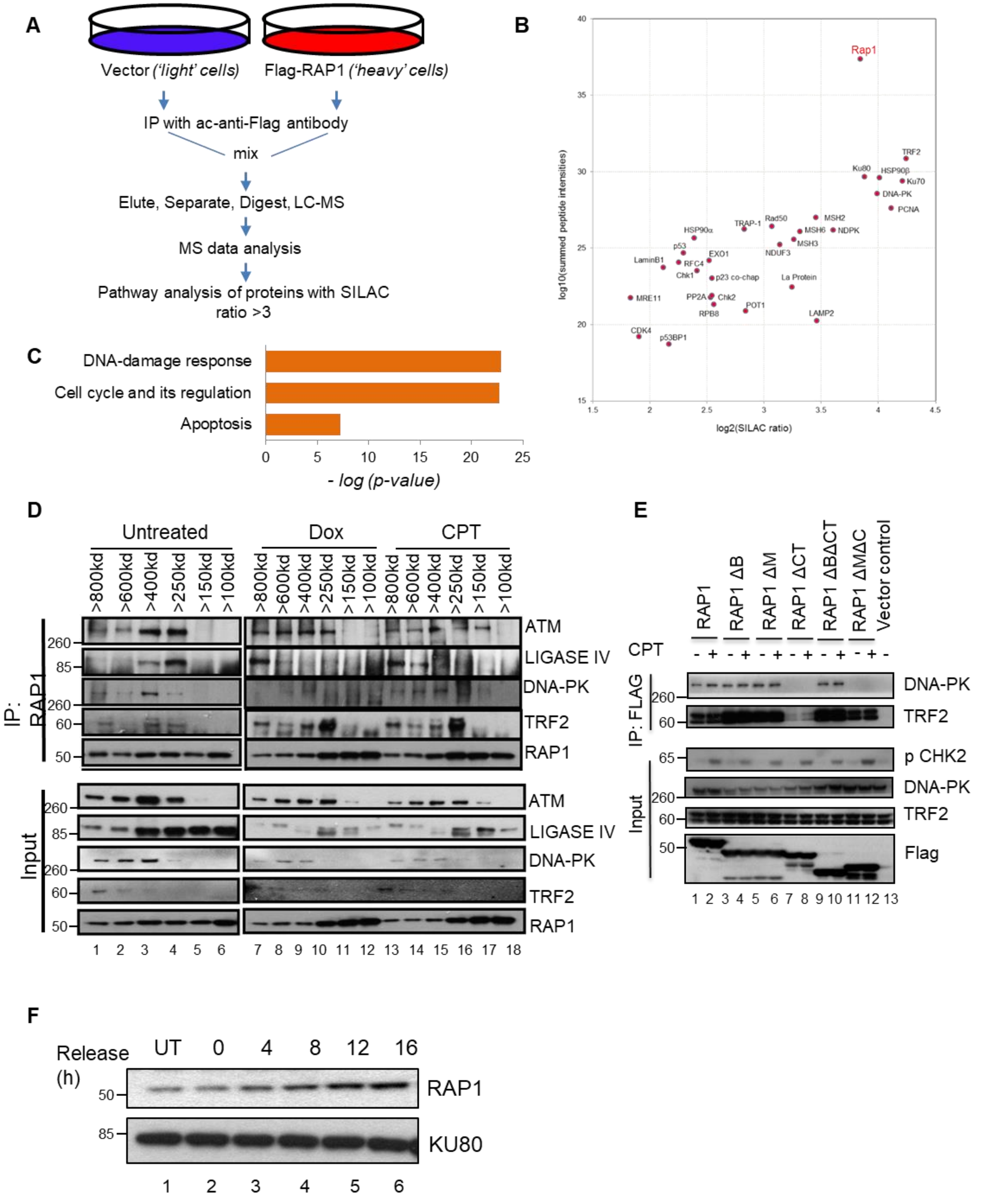

Supplementary Fig. 4. RAP1 interacts with members of the DNA damage response pathway.
(A) Identification of Rap1 interactome using SILAC-based quantitative proteomics. Protein complexes associated with RAP1 were identified by SILAC-based immunoprecipitaion (IP) of Flag-RAP1 expressing cells followed by high resolution mass spectrometry analysis. (B) Pathway analysis of proteins pulled down by RAP1 with SILAC ratios greater than 3 using GeneGO (MetaCore^{TM}, version 6.11). (C) Ratio-intensity plot of DNA-damage response protein cluster. Several DNA damage response proteins associated with RAP1 were identified by pathway analysis of SILAC data of Rap1 IP. (D) Large scale size exclusion chromatography and RAP1 IP of untreated cells and cells treated with indicated genotoxic drugs. TRF2 was immunoblotted as a positive control for RAP1 IP. (E) Domains of RAP1 required for binding to different DNA damage pathway members were mapped. Flag-tagged RAP1 mutants were overexpressed in cells followed by IP using anti-Flag beads. The IP eluate was immunoblotted for the indicated proteins. (F) Cells were treated with CPT (10μM) for 3h, released and fractionated to obtain nuclear extract. These nuclear extracts were western blotted for indicated proteins. UT refers to untreated sample.

Supplementary Figure 5

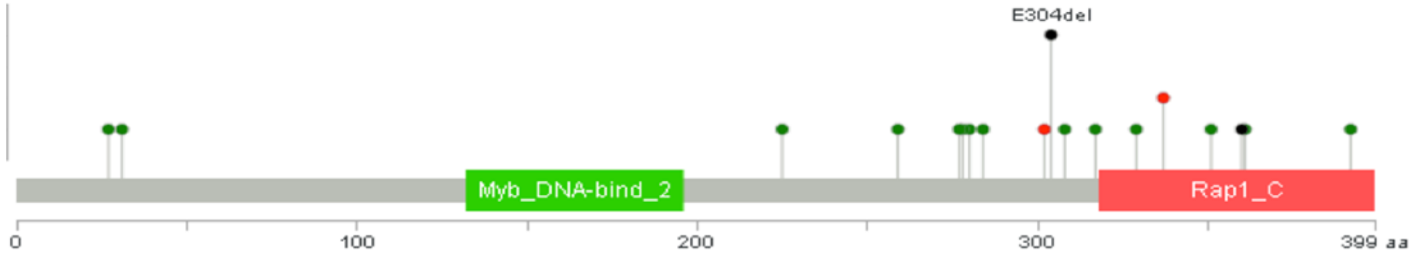

Supplementary Fig. 5. Novel Rap1 mutations in human cancers. Identified mutations in RAP1 in human cancer patients. Red and green dots represent nonsense and missense mutations in various cancers.

Supplementary Figure 6

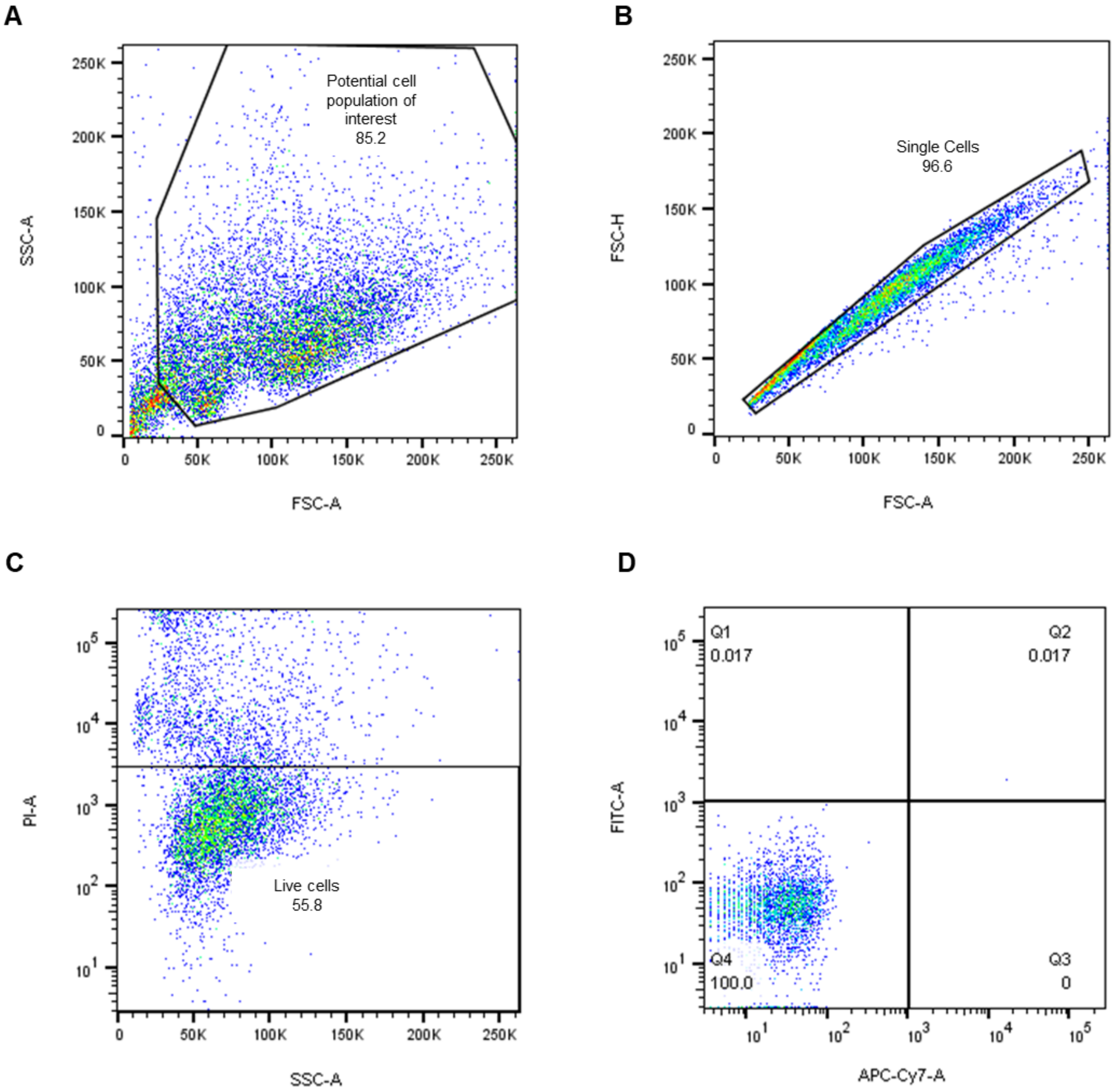

Supplementary Fig. 6. General gating strategy used

(A) Preliminary gating with SSC-A / FSC-A was used to gate out cell debris from potential cell population of interest. (B) FSC-H / FSC-A was used to gate for single cell population. (C) In most of the experiments, propidium iodide was used as a live-dead stain to gate for live cells. (D) Unstained cells were used to determine the gating for positively stained cells.

Supplementary Table 1. List of primers

Primer Name	Sequence	Remarks
shRap1_KD1_FW	CCGGgagagttcttgcattggaactCTCGAGagttccaatgcaagaactctcTTTTTG	shRNA of Rap1 construct 1
shRap1_KD1_RV	AATTCAAAAAgagagttcttgcattggaactCTCGAGagttccaatgcaagaactctc	shRNA of Rap1 construct 1
shRap1_KD2_FW	CCGGaactccagatttgcctgaagaCTCGAGtcttcaggcaaatctggagttTTTTTG	shRNA of Rap1 shRNA construct 2
shRap1_KD2_RV	AATTCAAAAAaactccagatttgcctgaagaCTCGAGtcttcaggcaaatctggagtt	shRNA of Rap1 shRNA construct 2
shControl_FW	CCGGCCTAAGGTTAAGTCGCCCTCGCTCGAGCGAGGGCGACTTAACCTTAGGTTTTTG	shRNA of scrambled Control construct
shControl_RV	AATTCAAAAACCTAAGGTTAAGTCGCCCTCGCTCGAGCGAGGGCGACTTAACCTTAGG	shRNA of scrambled Control construct
Rap1_271-276_FW	GTGGATGAGAGCCCTAATGCTGCTATAAGATCTATAACTATGTGTGATG	Linker mutation of Rap1 (p.271-276)
Rap1_271-276_RV	CATCACACATAGTTATAGATCTTATAGCAGCATTAGGGCTCTCATCCAC	Linker mutation of Rap1 (p.271-276)
Rap1_277-282_FW	GATTTTGAAATACATAACGCTGCGATTCGTAGTGATCCACCCACACCTG	Linker mutation of Rap1 (p.277-282)
Rap1_277-282_RV	CAGGTGTGGGTGGATCACTACGAATCGCAGCGTTATGTATTTCAAAATC	Linker mutation of Rap1 (p.277-282)
Rap1_283-288_FW	ACTATGTGTGATGATAATGCAGCCATACGTTCGGAAGACTCAGAAACAC	Linker mutation of Rap1 (p.283-288)
Rap1_283-288_RV	GTGTTTCTGAGTCTTCCGAACGTATGGCTGCATTATCATCACACATAGT	Linker mutation of Rap1 (p.283-288)
Rap1_289-294_FW	CCACCCACACCTGAGAACGCCGCAATAAGATCGCCTGATGAGGAGGAAG	Linker mutation of Rap1 (p.289-294)
Rap1_289-294_RV	CTTCCTCCTCATCAGGCGATCTTATTGCGGCGTTCTCAGGTGTGGGTGG	Linker mutation of Rap1 (p.289-294)
Rap1_295-300_FW	GAGGAAGACTCAGAAACACAGAATGCTGCGATCAGATCAGAAGAAGAAGAAAAAGTTTCTC	Linker mutation of Rap1 (p.295-300)
Rap1_295-300_RV	GAGAAACTTTTTCTTCTTCTTCTGATCTGATCGCAGCATTCTGTGTTTCTGAGTCTTCCTC	Linker mutation of Rap1 (p.295-300)
Rap1_301-306_FW	GATGAGGAGGAAGAAAACGCAGCAATAAGAAGTTCTCAACCAGAGGTGG	Linker mutation of Rap1 (p.301-306)
Rap1_301-306_RV	CCACCTCTGGTTGAGAACTTCTTATTGCTGCGTTTTCTTCCTCCTCATC	Linker mutation of Rap1 (p.301-306)
Rap1_307-312_FW	GAAGAAGAAAAAGTTAATGCAGCAATCCGGAGCGCTGCCATTAAGATC	Linker mutation of Rap1 (p.307-312)
Rap1_307-312_RV	GATCTTAATGGCAGCGCTCCGGATTGCTGCATTAACTTTTTCTTCTTC	Linker mutation of Rap1 (p.307-312)
Rap1_313-318_FW	CAACCAGAGGTGGGAAATGCCGCTATCCGCAGTCGGCAGTTAATGGAG	Linker mutation of Rap1 (p.313-318)
Rap1_313-318_RV	CTCCATTAACTGCCGACTGCGGATAGCGGCATTTCCCACCTCTGGTTG	Linker mutation of Rap1 (p.313-318)
Rap1_319-324_FW	GGAGCTGCCATTAAGATCATTAACGCGGCAATCCGGTCGTTTAACTTGGATCTATCAAC	Linker mutation of Rap1 (p.319-324)
Rap1_319-324_RV	GTTGATAGATCCAAGTTAAACGACCGGATTGCCGCGTTAATGATCTTAATGGCAGCTCC	Linker mutation of Rap1 (p.319-324)
Rap1_325-330_FW	CAGTTAATGGAGAAGAATGCCGCGATTCGATCAACAGTTACACAGGCC	Linker mutation of Rap1 (p.325-330)
Rap1_325-330_FW	GGCCTGTGTAACTGTTGATCGAATCGCGGCATTCTTCTCCATTAACTG	Linker mutation of Rap1 (p.325-330)
mRap1_FW	GCGGATAGCGGGGAACCACA	qPCR of mouse Rap1
mRap1_RV	CCGGGTGGCTTCCACAAGCAT	qPCR of mouse Rap1
AG45	CTCAGCCACTTATCCTATCCTGC	Genotyping
AG45	GCACAGAACTGATGCAGACG	Genotyping
hRAP1_FW	CCGCGGATAGCGGGGAACCA	qPCR of human RAP1
hRAP1_RV	ACTCCCGGGTGGCTTCCACAA	qPCR of human RAP1

*FW – Forward primer; RV – Reverse primer; p. – Amino acid
