# Supplementary material for: Rap1 regulates hematopoietic stem cell survival and affects oncogenesis and response to chemotherapy
Source: Nat Commun. 2019 Dec 13;10:5349. doi: 10.1038/s41467-019-13082-9 (PMC6911077; doi:10.1038/s41467-019-13082-9)
Supplement: Supplementary file 2 — Reporting Summary [file 41467_2019_13082_MOESM2_ESM.pdf]

nature research

Corresponding author(s): Vinay Tergaonkar

Last updated by author(s): Sep 26, 2019

Reporting Summary

Nature Research wishes to improve the reproducibility of the work that we publish. This form provides structure for consistency and transparency in reporting. For further information on Nature Research policies, see Authors & Referees and the Editorial Policy Checklist.

Statistics

For all statistical analyses, confirm that the following items are present in the figure legend, table legend, main text, or Methods section.

n/a	Confirmed	
☐	☒	The exact sample size (n) for each experimental group/condition, given as a discrete number and unit of measurement
☐	☒	A statement on whether measurements were taken from distinct samples or whether the same sample was measured repeatedly
☐	☒	The statistical test(s) used AND whether they are one- or two-sided<br>*Only common tests should be described solely by name; describe more complex techniques in the Methods section.*
☒	☐	A description of all covariates tested
☒	☐	A description of any assumptions or corrections, such as tests of normality and adjustment for multiple comparisons
☐	☒	A full description of the statistical parameters including central tendency (e.g. means) or other basic estimates (e.g. regression coefficient) AND variation (e.g. standard deviation) or associated estimates of uncertainty (e.g. confidence intervals)
☒	☐	For null hypothesis testing, the test statistic (e.g. F, t, r) with confidence intervals, effect sizes, degrees of freedom and P value noted<br>*Give P values as exact values whenever suitable.*
☒	☐	For Bayesian analysis, information on the choice of priors and Markov chain Monte Carlo settings
☒	☐	For hierarchical and complex designs, identification of the appropriate level for tests and full reporting of outcomes
☒	☐	Estimates of effect sizes (e.g. Cohen's d, Pearson's r), indicating how they were calculated

Our web collection on statistics for biologists contains articles on many of the points above.

Software and code

Policy information about availability of computer code

Data collection	No codes were used in data collection.
Data analysis	No codes were used in data analysis.

For manuscripts utilizing custom algorithms or software that are central to the research but not yet described in published literature, software must be made available to editors/reviewers. We strongly encourage code deposition in a community repository (e.g. GitHub). See the Nature Research guidelines for submitting code & software for further information.

Data

Policy information about availability of data

All manuscripts must include a data availability statement. This statement should provide the following information, where applicable:

- Accession codes, unique identifiers, or web links for publicly available datasets
- A list of figures that have associated raw data
- A description of any restrictions on data availability

Accession codes and unique identifiers have been provided in the Methods Section for publicly available colorectal cancer datasets (GSE17538 & GSE17538). A list of figures and supplementary figures that have associated raw data has been provided in the Source Data file. No restrictions have been put on data availability.

Field-specific reporting

Please select the one below that is the best fit for your research. If you are not sure, read the appropriate sections before making your selection.

☒ Life sciences ☐ Behavioural & social sciences ☐ Ecological, evolutionary & environmental sciences

Life sciences study design

All studies must disclose on these points even when the disclosure is negative.

Sample size	Sample size was determined based on the minumum number of animals / replicates that would be required to do power calculations.
Data exclusions	No data was excluded from the calculations unless the animals died during the course of the experiment.
Replication	All experiments were repeated at least twice to ensure reproducibility.
Randomization	For mice experiments, littermates were used. WT and knockout mice were randomly assigned to treatment or non-treatment groups.
Blinding	Blinding was not performed since it was not necessary for this study.

Reporting for specific materials, systems and methods

We require information from authors about some types of materials, experimental systems and methods used in many studies. Here, indicate whether each material, system or method listed is relevant to your study. If you are not sure if a list item applies to your research, read the appropriate section before selecting a response.

Materials & experimental systems

n/a	Involved in the study	
☐	☒	Antibodies
☐	☒	Eukaryotic cell lines
☒	☐	Palaeontology
☐	☒	Animals and other organisms
☒	☐	Human research participants
☒	☐	Clinical data

Methods

n/a	Involved in the study	
☒	☐	ChIP-seq
☐	☒	Flow cytometry
☒	☐	MRI-based neuroimaging

Antibodies

Antibodies used

All antibodies used are listed with the respective catalogue numbers in the Methods Section of the manuscript. Antibodies against Chk2 (Cell Signaling Technology: #2662), pChk2 (Cell Signaling Technology: #2661), ATM (Cell Signaling Technology: #2873S), and KU80 (Cell Signaling Technology: #2180) were from Cell Signaling Technology. Rap1 (Santa Cruz: sc-53434 and sc-28197) was from Santa Cruz Biotechnology, TRF2 (Millipore: #05-521) and pATM (Millipore: #05-740) antibody were from Millipore. Ligase IV (Proteintech: #12695-1-AP) antibody was from Proteintech. XRCC4 (ab145) and DNA-PK (ab1832) antibodies were from Abcam and used at 1:2000 dilutions. All other antibodies were used at 1:1000 dilutions. Anti-γ-H2AX antibody (Millipore: 05-636) and anti-53BP1 antibody (Cell Signaling Technology: #4937S) were used at 1:1000 dilution for immunofluorescence assays.

Hematopoietic stem and progenitor cell and lineage positive cell profiling was perform on bone marrow, spleen and thymus of mice from each genotype. Antibodies (clones) used: CD3 (145-2C11) [BD: #553064], CD4 (H129.19) [BD: #553653], CD8 (53-6.7) [BD: #553033], Gr-1 (RB6-8C5) [BD: #553128], B220 (RA3-6B2) [BD: #553090], Ter119 (Ter119) [BD: #553673], Mac1 (M1/70) [BD: #553311], IL7Rα (A7R34) [eBiosceinces: #12-1271-82], cKit (2B8) [BD: #558163], Sca-1 (E13-161.7) [eBiosceinces: #17-5981-83], CD25 (7D4) [eBiosciences: 13-0252-82],and CD44 (IM7) [BD: 559250], CD43 (S7) [BD: 561856], HSA (M1/69) [BD: #553262] and BP-1 (6C3) [BD: #553159], CD4 (GK1.5) [BD: #561830], CD8 (7D4) [eBiosciences: #12-0081-82], B220 (RA3-6B2) [BD: #561880].

Validation

All anitbodies used have been validated by their respective manufacturers' catalogue files to be compatible.

Eukaryotic cell lines

Policy information about cell lines

Cell line source(s)	All eukaryotic cell lines used in this study were originally obtained from ATCC and their catalogue numbers are listed in the Methods Section of the manuscript (MCF7 cells (ATCC® HTB-22™) and 293T (ATCC® CRL-3216™)).
Authentication	None of the cell lines were authenticated.
Mycoplasma contamination	All cell lines used in the study were negative for mycoplasma contamination.
Commonly misidentified lines (See ICLAC register)	None of the cell lines used in the study were commonly misidentified lines listed in the ICLAC

Animals and other organisms

Policy information about studies involving animals; ARRIVE guidelines recommended for reporting animal research

Laboratory animals	Mice
Wild animals	This study did not involve wild animals.
Field-collected samples	This study did not involve samples collected from the field.
Ethics oversight	Agency for Science, Technology and Research Singapore's Biological Resource Centre (BRC)'s Institutional Animal Care and Use Committee.

Note that full information on the approval of the study protocol must also be provided in the manuscript.

Flow Cytometry

Plots

Confirm that:

☒ The axis labels state the marker and fluorochrome used (e.g. CD4-FITC).

☒ The axis scales are clearly visible. Include numbers along axes only for bottom left plot of group (a 'group' is an analysis of identical markers).

☒ All plots are contour plots with outliers or pseudocolor plots.

☒ A numerical value for number of cells or percentage (with statistics) is provided.

Methodology

Sample preparation	Detailed sample preparation for each experiment is presented in the Methods Section of the manuscript.
Instrument	Instrument used for data collection is described in the Methods Section of the manuscript
Software	Software used for data analyses is described in the Methods Section of the manuscript
Cell population abundance	This data has been presented for all post-sort fractions.
Gating strategy	FSC-A/SSC-A gates were used as a preliminary gate for potential live cells and to exclude cell debris. The live cells were then gates with FSC-A/FSC-H to isolate single cell populations. An unstained sample was used to gate the negative population.

☒ Tick this box to confirm that a figure exemplifying the gating strategy is provided in the Supplementary Information.
